# *S*-nitrosylation triggers ABI5 degradation to promote seed germination and seedling growth

**DOI:** 10.1038/ncomms9669

**Published:** 2015-10-23

**Authors:** Pablo Albertos, María C. Romero-Puertas, Kiyoshi Tatematsu, Isabel Mateos, Inmaculada Sánchez-Vicente, Eiji Nambara, Oscar Lorenzo

**Affiliations:** 1 Dpto. de Microbiología y Genética, Instituto Hispano-Luso de Investigaciones Agrarias (CIALE), Facultad de Biología, Universidad de Salamanca, C/ Río Duero 12, Salamanca 37185, Spain; 2 Dpto. de Bioquímica, Biología Celular y Molecular de Plantas, Estación Experimental del Zaidín, Consejo Superior de Investigaciones Científicas, Profesor Albareda 1, Granada E-18008, Spain; 3 Laboratory of Plant Organ Development, National Institute for Basic Biology, Nishigonaka 38, Myodaiji, Okazaki 444-8585, Japan; 4 Department of Cell and Systems Biology, University of Toronto, 25 Willcocks Street, Toronto, Ontario, Canada M5S3B2

## Abstract

Plant survival depends on seed germination and progression through post-germinative developmental checkpoints. These processes are controlled by the stress phytohormone abscisic acid (ABA). ABA regulates the basic leucine zipper transcriptional factor ABI5, a central hub of growth repression, while the reactive nitrogen molecule nitric oxide (NO) counteracts ABA during seed germination. However, the molecular mechanisms by which seeds sense more favourable conditions and start germinating have remained elusive. Here we show that ABI5 promotes growth via NO, and that ABI5 accumulation is altered in genetic backgrounds with impaired NO homeostasis. *S*-nitrosylation of ABI5 at cysteine-153 facilitates its degradation through CULLIN4-based and KEEP ON GOING E3 ligases, and promotes seed germination. Conversely, mutation of ABI5 at cysteine-153 deregulates protein stability and inhibition of seed germination by NO depletion. These findings suggest an inverse molecular link between NO and ABA hormone signalling through distinct posttranslational modifications of ABI5 during early seedling development.

## Abstract (web summary)

Nitric oxide counteracts the inhibitory effects of the plant hormone ABA during seed germination and seedling growth. Here, Albertos *et al*. show that nitric oxide can act antagonistically to ABA by inducing the *S*-nitrosylation and degradation of the ABI5 transcription factor involved in ABA signalling.

## Graphical abstract

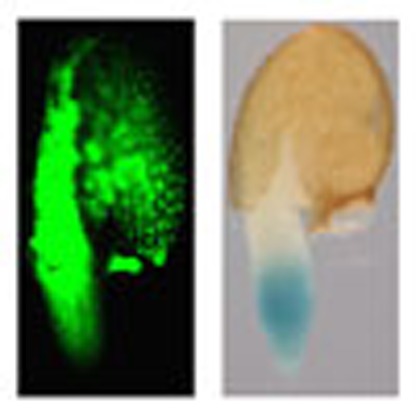

In the plant life cycle, the development of a new seedling depends on both appropriate timing of seed germination and the perception of environmental conditions. Consequently, germination and early seedling development must be tightly regulated by exogenous and endogenous signal molecules. Among them, abscisic acid (ABA) plays an important role in the inhibition of seed germination^1^ and in post-germinative seedling arrest under unfavourable environmental conditions^2^. ABA-dependent growth arrest after germination relies on the basic leucine zipper-type transcription factor ABI5 (refs 3, 4, 5). In many plant developmental processes and stress responses, the precise changes in cellular status are regulated not solely by ABA signalling but by a complex network of ABA and other signalling pathways. Several examples of cross-talk between ABA and the gaseous signalling molecule nitric oxide (NO) have been recently reported. These interactions include the link of SnRK2.6/OST1 *S*-nitrosylation to the negative regulation of ABA signalling in the stomata^6^ and the NO regulation of *ABI5* transcription through control of group VII ethylene response factors (ERFs) stability^7^. NO is a signalling molecule involved in a variety of physiological processes during plant growth and development. Extensive research has shown that NO affects seed dormancy, seed germination and ABA sensitivity^8^,^9^, as evidenced by exogenous application of NO donors^10^ or by genetic analysis of mutants with altered endogenous NO levels in *Arabidopsis*^11^. However, despite the abundant involvement of NO in different plant cell signalling pathways, the actual knowledge about its direct targets is poorly understood. A key feature of NO biology is the posttranslational modification of cysteine thiol to form nitrosothiols (*S*-nitrosylation) in target proteins^12^. In animals, this posttranslational modification has also been related to protein degradation via the ubiquitin-dependent proteasome pathway^13^. Here we establish a molecular mechanism for NO and ABA antagonism in the regulation of seed germination and post-germinative development through ABI5 protein stability. Genetic analysis identified that *abi5* mutants are insensitive to NO scavenging during seed germination. ABI5 protein levels are high in NO-deficient mutant backgrounds and low in NO-overaccumulating plants. *S*-nitrosylation of ABI5 at Cys 153 facilitates its degradation and promotes seed germination. Conversely, mutation of ABI5 Cys 153 reduces protein degradation through CULLIN4 (CUL4)-based and KEEP ON GOING E3 (KEG) ligases, and deregulates the inhibition of seed germination by NO depletion. Thus, ABI5 is regulated through the antagonistic action of ABA and NO, as evidenced by the synergistic effect of *S*-nitrosoglutathione (GSNO) on ABI5 destabilization. These findings suggest an inverse molecular link between NO and ABA hormone signalling through distinct posttranslational modifications of ABI5 during gene regulation of early seedling development.

## Results

### Identification and genetic characterization of *gap* mutants

**Figure 1:**
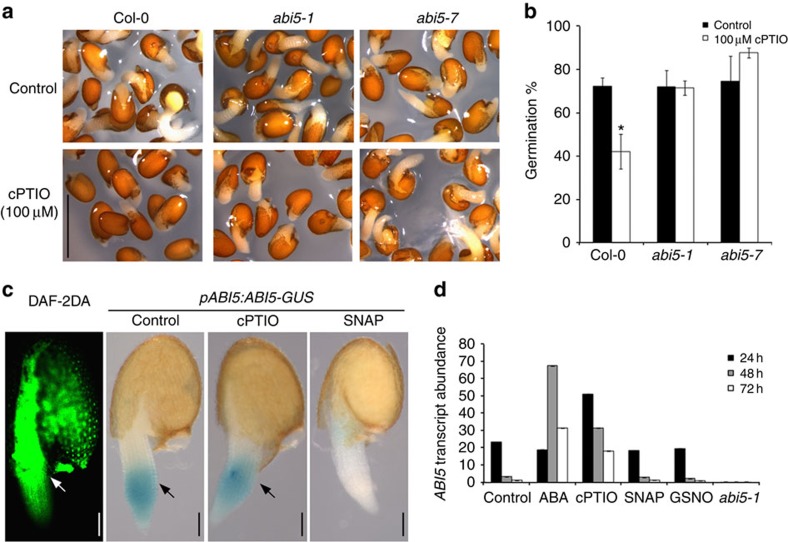
NO depletion phenotypes of ABI5 loss-of-function mutants during seed germination. (**a**) Insensitivity of *abi5* mutants to NO scavenging by cPTIO during seed germination. Photographs of 2-day-old germinated seeds after imbibition of wild type (Col-0) and the ABA-insensitive *abi5-1* and *abi5-7* mutants, in the absence of (Control) or the presence of 100 μM cPTIO. Scale bar, 1 mm. (**b**) Germination of wild-type (Col-0), *abi5-1* and *abi5-7* seeds in media containing 0 and 100 μM cPTIO after 2 days. Error bars represent±s.e. (*n*=3). Asterisk indicates significant differences compared with Col-0 (Control) (*t*-test, *P*<0.05). (**c**) Co-localization of ABI5 expression, protein localization and NO production. *pABI5:ABI5-GUS* seeds were stratified for 3 days at 4 °C and grown for 1 to 2 days at 21 °C on MS agar plates and then subjected to DAF-2DA incubation or GUS staining after treatment with NO scavenger (cPTIO) and donor (SNAP). Arrows indicate high NO accumulation (left), and ABI5 expression and protein localization (middle). Scale bars, 100 μm. (**d**) qRT–PCR analysis of *ABI5* relative transcript abundance in Col-0 seeds untreated (Control) and after treatments with ABA, cPTIO, SNAP and GSNO after 24, 48 and 72 h and in the *abi5-1* background. Error bars represent±s.e. (*n*=3).

NO affects seed dormancy, seed germination and ABA sensitivity^8^,^9^, as shown by exogenous application of NO donors^10^ or by genetic analysis of mutants with altered endogenous NO levels in *Arabidopsis*^11^. By exploiting the cross-talk between ABA and NO in the transition from dormancy to germination, we performed a genetic screen using (+)-*S*-ABA coupled to the effect of the NO scavenger 2-(4-carboxyphenyl)-4,4,5,5-tetramethylimidazoline-1-oxyl-3-oxide (cPTIO). We isolated 7 (+)-*S*-ABA-insensitive mutants that were able to germinate on 3 μM (+)-*S*-ABA and also display a cPTIO-insensitive phenotype (Supplementary Fig. 1a). These mutants were named *gap* (*germination in ABA and cPTIO*). Allelism tests indicated that these fell into two different loci, five of these mutants corresponding to new *abi5* alleles verified by candidate gene sequencing (Supplementary Fig. 1b). Germination of Col-0 wild-type seeds was delayed by 100 μM cPTIO (Fig. 1a,b), in agreement with previous reports^9^; however, germination of the *abi5* mutant alleles was less affected by NO depletion than the wild type. Hence, these results showed that *abi5* mutants were insensitive not only to ABA but also to NO scavenging by cPTIO during seed germination.

### NO induces degradation of the ABI5 protein

To extend these findings, we determined the localization of the ABI5 protein (Fig. 1c) and *ABI5* transcript (Fig. 1d) in seeds after treatment with ABA, the NO donor *S*-nitroso-*N*-acetyl-DL-penicillamine (SNAP) and the NO scavenger cPTIO. *ABI5* accumulated to high levels after treatment with ABA for 48 or 72 h and even earlier after NO was depleted (Fig. 1d). In contrast, application of the NO donor SNAP quickly reduced ABI5-GUS levels (Fig. 1c and Supplementary Fig. 2).

To further investigate the role of NO in the regulation of seed germination in *Arabidopsis*, we examined endogenous NO levels in 2-day-old wild-type seeds by using the fluorescence indicator 4,5-diaminofluorescein diacetate (DAF-2DA). We observed NO-dependent fluorescence in the seed endosperm and rapid elongation zone after radicle protrusion (Fig. 1c), as previously described^14^,^15^. Application of the NO scavenger cPTIO reduced the DAF-2DA fluorescence pattern in treated seeds, signal being limited to the autoflorescence of the testa tissues (Supplementary Fig. 1c–e). Co-localization of NO burst and ABI5 tissue-specific reduction of ABI5-GUS protein during radicle protrusion (seed imbibition/germination) was confirmed after GUS histochemical analysis using *pABI5:ABI5-GUS* lines as highlighted in Fig. 1c.

**Figure 2:**
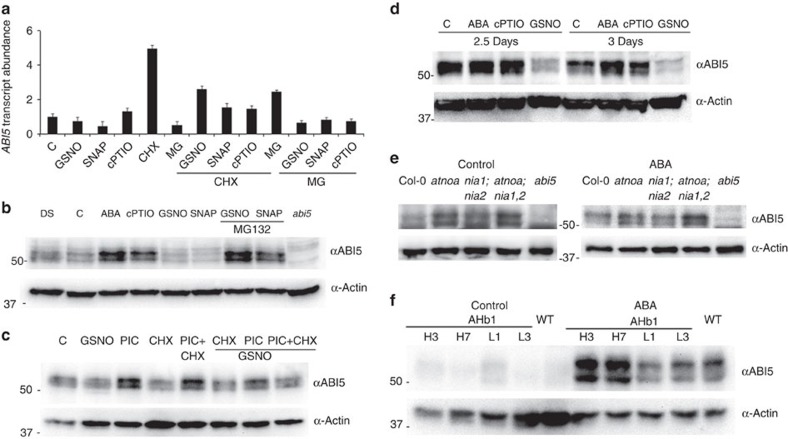
Increased NO levels reduce ABI5 protein accumulation in a proteasome pathway-dependent manner. (**a**) qRT–PCR analysis of *ABI5* relative transcript abundance in Col-0 seeds untreated (C) and after treatments with GSNO, SNAP, cPTIO, cycloheximide (CHX), MG132 proteasome inhibitor and the combinations indicated after 3 h. Error bars represent±s.e. (*n*=3). (**b**) SNAP and GSNO treatments promote ABI5 degradation in dormant seeds (DS). Immunoblot analysis of ABI5 protein levels in seed extracts of Col-0 and *abi5-1*, treated with or without (C) NO scavenger (cPTIO) and donors (GSNO and SNAP), and the MG132 proteasome inhibitor. Actin protein levels are shown as a loading control. (**c**) Immunoblot analysis of ABI5 protein levels in seed extracts of Col-0 dormant seeds treated with or without (C) NO donor (GSNO), the proteasome inhibitor cocktail (PIC, composed of MG115, MG132 and epoxomicin), cycloheximide (CHX) and the combinations indicated. Actin protein levels are shown as a loading control. (**d**) GSNO treatment promotes ABI5 degradation in 2 days ABA (5 μM)-treated after-ripened seeds. Immunoblot analysis of ABI5 protein levels in seed extracts of Col-0, treated with or without (C) NO scavenger (cPTIO) and donor (GSNO). Actin protein levels are shown as a loading control. (**e**) ABI5 protein levels in wild type (WT; Col-0), *abi5-1* and NO-deficient (*atnoa1-1, nia1;nia2, atnoa1-2;nia1;nia2)* mutant backgrounds. Stratified seeds were sown on control MS (Control, left) and 0.1 μM ABA after 48 h (right). Immunoblot analysis of ABI5 protein levels in seed extracts of WT and NO-deficient mutants. Actin protein levels are shown as a loading control. (**f**) ABI5 protein levels in WT, *35S:AHb1* (H3, H7) and *35S:antiAHb1* (L1, L3) lines. Stratified seeds were sown on MS (Control) and 0.1 μM ABA for 96 h. Immunoblot analysis of ABI5 protein levels in seed extracts of AHb1-overexpressing and -silencing lines. Actin protein levels are shown as a loading control.

Recently, the N-end rule pathway of targeted proteolysis has been shown to regulate *ABI5* expression through group VII ERF transcription factors^7^. To this end, application of MG132 did not transcriptionally induce *ABI5* gene expression, as a putative consequence of enhanced group VII ERF stabilization (Fig. 2a).

The effect of SNAP and the physiological NO donor GSNO in the promotion of seed germination was correlated by the disappearance of ABI5 during germination and post-germinative growth (Fig. 2b–d and Supplementary Fig. 3a–c). Conversely, endogenous NO depletion by the cPTIO scavenger inhibited seed germination and maintained high ABI5 protein levels, similar to ABA (Fig. 2b,d). It is noteworthy that the proteasome inhibitor MG132 or the proteasome inhibitor cocktail (including MG115, MG132 and epoxomicin) restored ABI5 accumulation, even in the presence of NO donors, and prevented seed germination (Fig. 2b,c and Supplementary Fig. 3c). In addition, cycloheximide alongside the MG132 and NO donor/scavenger treatments proved that ABI5 protein stability was being affected (Fig. 2a,c). Collectively, these findings implied that NO function during seed germination was through ABI5 degradation. After this developmental checkpoint and at high concentrations, NO dramatically affected post-germinative seedling growth, inhibiting root growth and development^15^.

### ABI5 level is altered when NO homeostasis is impaired

We corroborated the above pharmacological findings by the use of mutants and transgenic lines impaired in NO homeostasis. Thus, NO-deficient *atnoa1-2;nia1;nia2* triple mutant impaired in NIA/NR- and AtNOA1-dependent NO biosynthesis in *Arabidopsis* was hypersensitive to ABA, underscoring its effect on germination inhibition^11^ (Supplementary Fig. 3d). Non-symbiotic haemoglobin 1 (AHb1) is an endogenous scavenger of NO, and thus AHb1-overexpressing and -silenced lines contain lower and higher NO levels, respectively^16^. As expected, the ABA response phenotype of these AHb1 lines during seed germination differed from the hypersensitivity to ABA of the AHb1-overexpressing lines to the wild-type germination of the AHb1-silenced lines (Supplementary Fig. 3e,f).

We investigated ABI5 protein accumulation in the *Arabidopsis atnoa1, nia1;nia2* and *atnoa1;nia1;nia2* mutant seeds, which exhibit decreased levels of cellular NO^11^ (Fig. 2e). NO-deficient mutants, defective in either the oxidative or reductive NO synthesis pathways, respectively, or in both pathways, accumulated higher ABI5 protein levels. In agreement with the enhanced ABA sensitivity of NO-deficient mutant backgrounds, ABI5 protein levels were increased with respect to those observed in the wild type (Col-0). To corroborate these findings, AHb1-overexpressing and -silenced lines were also analysed for their ABI5 accumulation pattern (Fig. 2f). Collectively, these findings implied that genetic backgrounds where the endogenous NO levels were enhanced (AHb1-silencing lines) or diminished (*atnoa1*, *nia1;nia2*, *atnoa1;nia1;nia2* and AHb1-overexpressing lines) displayed altered ABI5 levels, which were mirrored by changes in their ABA response. These pharmacological and genetic approaches suggest that NO promoted ABI5 protein degradation via a proteasome-dependent pathway.

### ABI5 is *S*-nitrosylated *in vivo* and *in vitro*

**Figure 3:**
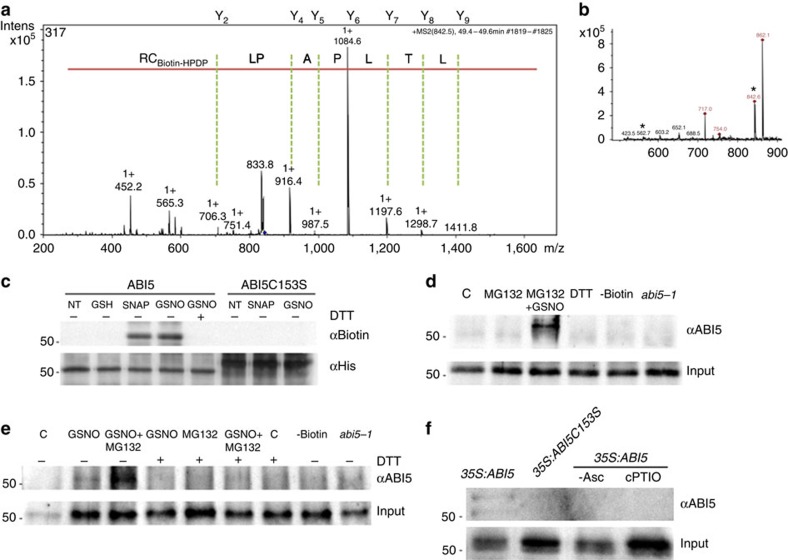
*S*-nitrosylation of ABI5 *in vivo* and *in vitro*. (**a**) Mass spectrometric analyses identify C153 as the *S*-nitrosylation site. MS/MS spectra of C153 from the tryptic fragment QGSLTLPAPLCR (peptide MS/MS spectra shown with Cys modified by biotin-HPDP). (**b**) The LC–MS spectra of the corresponding peaks (*562 *m/z* (3+) and 842,49 *m/z* (+2)) of this peptide fragment is shown in the inset. (**c**) The C153S mutation blocks *S*-nitrosylation of ABI5. *In vitro S*-nitrosylation of wild-type ABI5 and mutant ABI5C153S recombinant proteins by the NO donors GSNO (200 μM) and SNAP (200 μM). This modification is reversed by treatment with DTT (20 mM). No signal was observed with glutathione (200 μM) treatment showing specificity of the biotin-switch assay. ABI5 protein loading was detected by anti-His antibody. (**d**,**e**) *S*-nitrosylation of ABI5 induced by GSNO in after-ripened seed extracts. Samples were initially immunopurified with anti-biotin before immunoblot analysis of ABI5 protein levels in seed extracts of Col-0 (**d**), *35S:ABI5* (**e**) and *abi5-1* untreated (C) or treated with the indicated compounds. No signal was observed in the absence of biotin (−Biotin) or after DTT (20 mM) treatment. Actin protein levels are shown as a loading control. (**f**) *In-vivo S*-nitrosylation of ABI5 in *abi5-1;35S:cMyc-ABI5* and *abi5-1;35S:cMyc-ABI5C153S* after-ripened seed extracts 24 h after proteasome inhibitor MG132 (100 μM) incubation. Immunoblot analysis of *in vivo* ABI5 protein levels after immunopurification of *S*-nitrosylated proteins. No signal was observed in the absence of sodium ascorbate (−Asc) or after cPTIO (1 mM) treatment. Input protein levels were also determined using anti-ABI5 anti-serum.

One possible mechanism of NO action in plant tissues is the redox-based posttranslational modification of target proteins through *S*-nitrosylation. NO is able to reversibly modify thiol groups of specific cysteine residues in target proteins, hence altering protein function^12^. To determine whether ABI5 was *S*-nitrosylated by NO, the recombinant protein was exposed to either GSNO or SNAP, which are typically used to evaluate *S*-nitrosylation (that is, SNO formation) *in vitro*^17^. The formation of SNO-ABI5 was monitored by the biotin switch method^18^. As shown in Fig. 3a–c, ABI5 was *S*-nitrosylated by either GSNO or SNAP. Furthermore, the addition of dithiothreitol (DTT) strongly reduced the formation of SNO-ABI5, consistent with the presence of a reversible thiol modification. The biotin switch was assayed without ascorbate, to assure that the immune reactivity of the protein was ascorbate dependent (Supplementary Fig. 4a) and to prevent switching SNO for biotin, as the method describes^18^. ABI5 contains four cysteine residues (Cys 56, Cys 153, Cys 293 and Cys 440) that might serve as sites for this redox-based modification (Supplementary Fig. 4b). *In-silico* prediction suggested that Cys 153 was potentially *S*-nitrosylated (Supplementary Fig. 4d,e). Mass spectrometry analysis confirmed *S*-nitrosothiol formation at only Cys 153 of ABI5 (Fig. 3a,b and Supplementary Figs 5 and 6). This residue was therefore mutated individually and the resulting protein was expressed and treated with GSNO before analysis with the biotin switch method. The Cys153 to Ser mutation abolished *S*-nitrosylation of ABI5 (Fig. 3c). Collectively, these findings indicated that Cys 153 of ABI5 was specifically *S*-nitrosylated *in vitro*. This Cys 153 is only present in the closest ABI5 homologue AtbZIP67 (Supplementary Fig. 4c), sharing a seed expression pattern with *ABI5* (Supplementary Fig. 7). Together, these data suggest that redox modification by *S*-nitrosylation may regulate the activity of these b-ZIP transcription factors in plants to govern seed germination and seedling establishment.

To understand whether *S*-nitrosylation of Cys 153 could modulate the function of ABI5, we assessed the effect of this NO-mediated posttranslational modification on the previously reported homodimerization of the ABI5 protein^19^ by using a yeast two-hybrid assay. Interestingly, neither the ability of ABI5 to interact (Supplementary Fig. 8) nor the DNA-binding capacity to the ABRE *cis*-consensus motif (Supplementary Fig. 9) were disturbed by the Cys–Ser mutation or in the presence of NO-related compounds.

To determine whether ABI5 was *S*-nitrosylated *in vitro* during seed germination, protein extracts from wild-type and *35S:ABI5* transgenic plants were treated with GSNO and MG132, and assayed by the biotin switch method, then *S*-nitrosylated proteins were immunopurified (Fig. 3d,e). Protein gel blot analysis of purified proteins probed with an antibody specifically recognizing the ABI5 protein showed the corresponding *S*-nitrosylation *in vitro*. In addition, *in vivo* studies with transgenic ABI5 lines expressing either Myc-tagged wild-type ABI5 or mutant derivatives were analysed during seed germination and treatment with MG132. To this end, endogenous proteins were subjected to biotin-switch analysis, immunoprecipitated and detected with an anti-ABI5 antibody. Wild-type ABI5 was *S*-nitrosylated during seed germination, but the Cys153Ser mutant was not (Fig. 3f). Therefore, these results suggest that NO may regulate ABI5 redox state by *S*-nitrosylation at Cys 153 during seed imbibition.

### ABI5 is a NO sensor during seed germination

**Figure 4:**
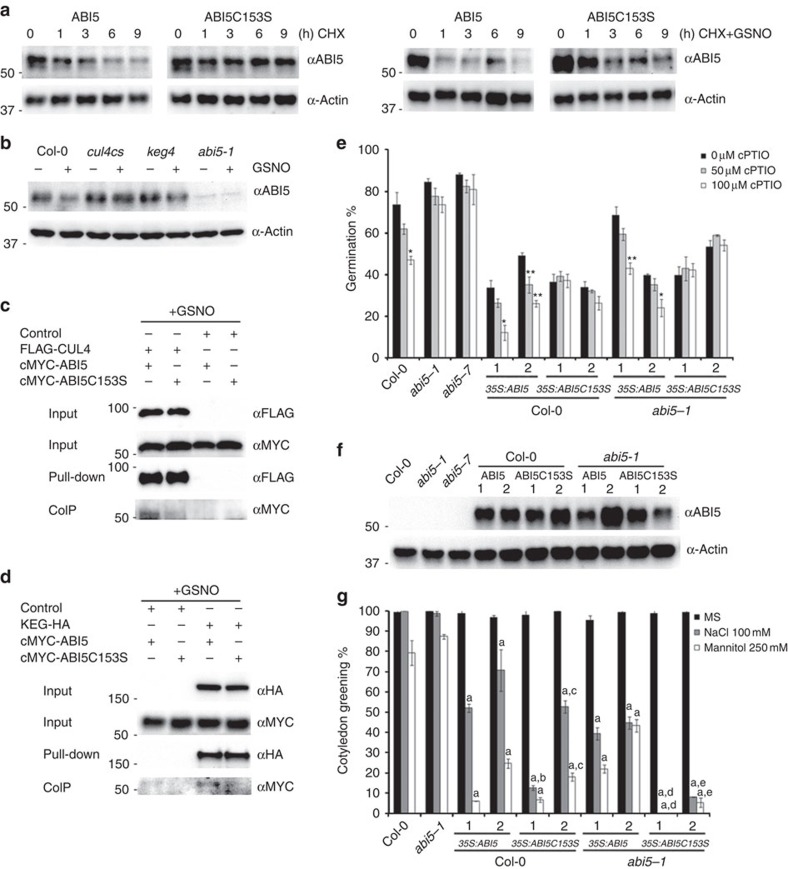
The ABI5 C153S mutant shows decreased proteasomal degradation by CUL4 and KEG, and confers NO insensitivity during seed germination. (**a**) Immunoblot analysis of ABI5 protein levels in 8-day-old seedling extracts of similar germination stages *abi5-1*;*35S:cMyc-ABI5* and *abi5-1*;*35S:cMyc-ABI5C153S*, in the presence of cycloheximide (1 mM) and cycloheximide (1 mM) plus GSNO (500 μM) from 0 to 9 h. Actin protein levels are shown as a loading control. (**b**) ABI5 protein levels in wild type (Col-0), *cul4cs*, *keg4* and *abi5-1* mutant backgrounds. Stratified seeds were incubated with 5 μM ABA for 48 h and treated with GSNO (1 mM) for 6 h after ABA removal. Immunoblot analysis of ABI5 protein levels in seed extracts of wild type and mutants. Actin protein levels are shown as a loading control. (**c**) Co-immunoprecipitation assays between CUL4 and transgenic ABI5/ABI5C153S proteins in the presence of GSNO. Input protein levels were also determined using anti-FLAG and anti-MYC antisera, respectively. (**d**) Co-immunoprecipitation assays between KEG and ABI5/ABI5C153S proteins in the presence of GSNO. Input protein levels were also determined using anti-HA and anti-MYC antisera, respectively. (**e**) NO-insensitive inhibition of seed germination to NO scavenging in *35S:ABI5C153S* lines as compared with *35S:ABI5* plants. Total seed germination of wild type (Col-0), *abi5-1*, *abi5-7* and two (1, 2) *35S:ABI5*- and *35S:ABI5C153S*-independent lines grown for 2 days on MS agar plates untreated (Control) or supplemented with 50 and 100 μM of the NO-scavenger cPTIO. Values represent the mean ±s.e. (*n*=3). Asterisks indicate significant differences compared with 0 μM cPTIO (*t*-test, *P<0.05, **P<0.01). (**f**) ABI5 levels in *35S:ABI5* and *35S:ABI5C153S* transgenic lines used for the germination assay. Immunoblot analysis of ABI5 protein levels in seed extracts. Actin protein levels are shown as a loading control. (**g**) NaCl- and mannitol-hypersensitive inhibition of post-germinative growth in two *35S:ABI5* and *35S:ABI5C153S* lines as compared with wild-type plants. Seedling growth of wild type (Col-0), *abi5-1*, *35S:ABI5* and *35S:ABI5C153S* lines grown for 9 days on MS agar plates untreated (Control) or supplemented with 100 mM of NaCl and 250 mM of mannitol. Values represent the mean±s.e. (*n*=3). Letters indicate significant differences compared with wild-type (Col-0) (a), *35S:ABI5*-1 (b), *35S:ABI5-2* (c), *abi5-1;35S:ABI5*-1 (e), *abi5-1;35S:ABI5*-2 (d), (*t*-test, *P*<0.05).

To assess a physiological role for *S*-nitrosylation of the ABI5 protein during seed germination, ABI5- and ABI5Cys153Ser-overexpressing lines were generated in *Arabidopsis* by expressing this protein under control of the 35S promoter, in the wild-type and *abi5-1* mutant background (Supplementary Fig. 10). The phenotype of the transgenic plants was evaluated in response to NO and ABA, and was compared with that of *abi5* mutant and *35S:ABI5* transgenic plants. Intact *ABI5*- and mutated *ABI5Cys153Ser*-overexpressing lines exhibited ABA-hypersensitive phenotypes during seed germination and seedling establishment (Supplementary Fig. 10d), demonstrating that ABI5Cys153Ser was able to largely restore the *abi5-1* mutation (Supplementary Fig. 10e). To further explore the possible biological consequence of ABI5-SNO formation at Cys 153, ABI5 protein accumulation was monitored in the presence of the protein synthesis inhibitor cycloheximide with or without GSNO (Fig. 4a). The data obtained implied that the mutation of ABI5 at Cys 153, considering similar seed germination stages, impaired NO-promoted ABI5 degradation, resulting in elevated levels of ABI5 protein. CUL4 and KEG are well-known factors that directly bind and regulate ABI5 ubiquitin-mediated proteolysis^20^,^21^,^22^,^23^,^24^. Accordingly, mutations in either CUL4 or KEG abolished NO-promoted ABI5 protein degradation (Fig. 4b and Supplementary Fig. 11). Intact ABI5 protein interacted with both CUL4 and KEG in the presence of GSNO, whereas ABI5Cys153Ser mutation failed to interact (Fig. 4c,d), supporting that *S*-nitrosylation triggered ABI5 destabilization through CUL4-based and KEG E3 ligases.

To determine the possible impact of the ABI5Cys153Ser mutation on NO sensing during seed germination and seedling establishment, we treated intact *ABI5*- and mutated *ABI5Cys153Ser*-overexpressing lines with NO scavengers (cPTIO) and donors (SNAP) (Fig. 4e,f and Supplementary Fig. 10f). As depicted in Fig. 4e,f, inhibition of seed germination by NO depletion during radicle protrusion was enhanced in *ABI5* but not in *ABI5Cys153Ser* overexpressors and also in the establishment of a new plant (Supplementary Fig. 10f), resulting in a prominent decrease in the rate of germination. In addition, there was a significant increase in seedling establishment after NO treatment in the *ABI5*-overexpressing lines relative to the overexpression of the *ABI5Cys153Ser* mutant version that instead was able to maintain growth arrest (Supplementary Fig. 10f). Germination of the transgenic plants under unfavourable conditions such as high salinity and hyperosmotic stress showed NaCl- and mannitol-hypersensitive inhibition of post-germinative growth in *35S:ABI5* and *35S:ABI5C153S* lines as compared with wild-type plants (Fig. 4g). Indeed, *35S:ABI5C153S* lines were more deeply hypersensitive than the *ABI5*-overexpressing lines. Together, this information implies that one way in which seeds sense NO was by the *S*-nitrosylation of ABI5 at Cys 153, and that disruption of this mechanism was able to arrest seedling growth under adverse environmental conditions.

**Figure 5:**
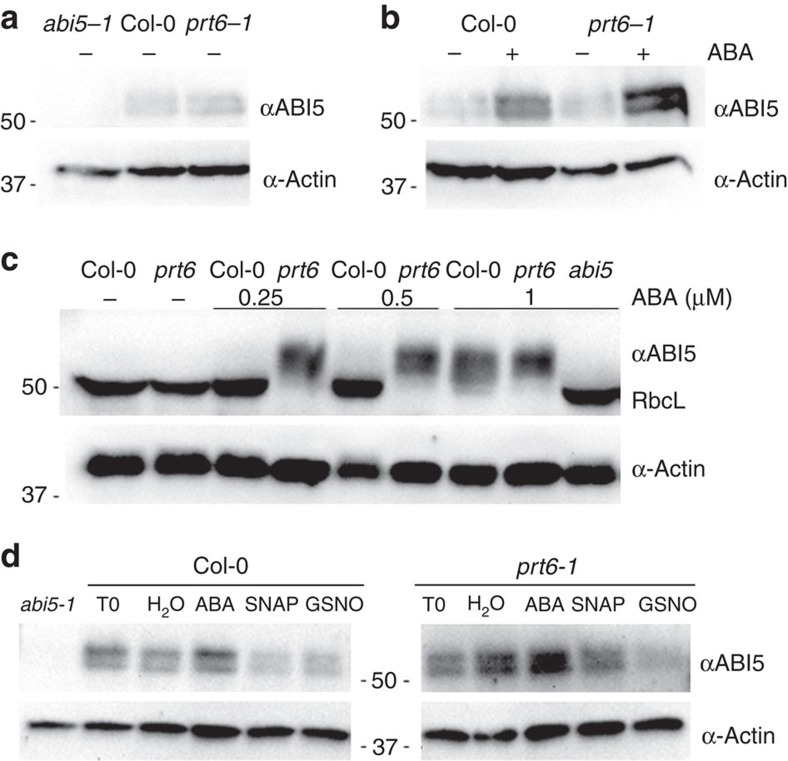
ABI5 accumulation and degradation in *prt6-1* mutant background during seed germination and post germination. (**a**,**b**) ABA treatment promotes ABI5 accumulation in germinating seeds. Immunoblot analysis of ABI5 protein levels in seed extracts of Col-0 and *prt6-1* before (**a**) and after 48 h treatment with (+) or without (−) 0.25 μM ABA (**b**). Actin protein levels are shown as a loading control. (**c**) Post-germinative ABI5 accumulation in seedlings. Immunoblot analysis of ABI5 protein levels in 10-day-old extracts of Col-0, *prt6-1* and *abi5-1*, treated with or without (−) 0.25, 0.5 and 1 μM ABA. Actin protein levels are shown as a loading control and Rubisco large subunit (RbcL) detection is indicated. (**d**) ABI5 protein levels in wild-type (Col-0), *prt6-1* and *abi5-1* mutant backgrounds. Stratified seeds were incubated with 5 μM ABA for 48 h (T0) and treated after ABA removal with H_2_O, ABA, SNAP (1 mM) and GSNO (1 mM) for 12 h. Immunoblot analysis of ABI5 protein levels in seed extracts of wild type and mutants. Actin protein levels are shown as a loading control.

The involvement of group VII ERF transcription factors in NO sensing and NO downregulation of *ABI5* transcription has been previously reported^7^. Consistently, ABI5 protein levels accumulated to a greater extent after ABA treatment in *prt6-1* mutant seeds and seedlings, which are impaired in the degradation of group VII ERFs by the N-end rule pathway^25^,^26^ (Fig. 5a–c). Thus, Rubisco large subunit was detected in those conditions/genotypes where the development of green and expanded cotyledons had taken place, producing seedlings with the ability to fix carbon during photosynthesis (Fig. 5c). The key role of ABA in the inhibition of seed germination and in post-germinative seedling arrest (that is, the absence of green and expanded cotyledons) was also highlighted, as no detection of Rubisco large subunit could be found in those ABA-hypersensitive genotypes (*prt6*) or in the Col-0 wild type under high ABA concentrations. Conversely, ABI5 was only detected in those conditions/genotypes where the seed germination was inhibited and or seedling growth arrested at post-germination developmental checkpoints. Here we also demonstrated that NO-induced ABI5 degradation by the proteasome could occur in a *prt6-1* mutant background (Fig. 5d). Thus, NO could promote ABI5 protein degradation independently of the NO-dependent transcriptional control of *ABI5* by the N-end rule pathway. Taken together, our results demonstrated a pivotal role of ABI5 in antagonistic ABA and NO signalling in the regulation of seed germination and highlighted ABI5 as a critical NO sensor in seeds.

## Discussion

**Figure 6:**
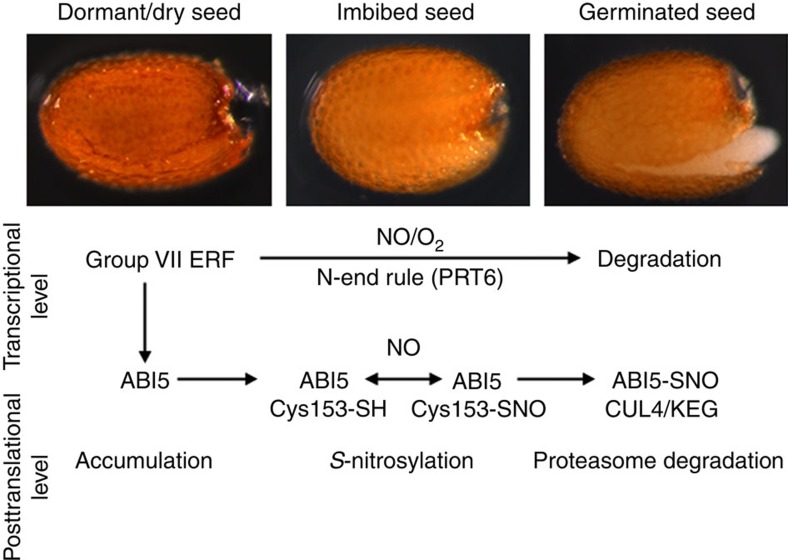
Model showing a role for NO in the regulation of ABI5 during seed germination. Transcriptional control of *ABI5* expression via the group VII ERFs^7^ and posttranslational *S*-nitrosylation of ABI5 protein are included. Dormant and dry seeds accumulate high levels of the ABA-induced ABI5 growth reppressor. On seed imbibition, a burst of NO is early produced to degrade group VII ERFs via the N-end rule pathway of targeted proteolysis (PRT6)^25^,^26^ and induces ABI5 *S*-nitrosylation promoting the interaction with CUL4-based and KEG E3 ligases. Consequently, ABI5 is rapidly degraded by the proteasome during seed germination.

In conclusion, our data establish a molecular mechanism for NO and ABA antagonism in the regulation of seed germination and post-germinative growth (Fig. 6). We identify two new loci involved in ABA and NO signalling (*GERMINATION IN ABA AND cPTIO*, *GAP*) and characterize one of these loci that correspond to the basic leucine zipper transcription activator ABI5. *S*-nitrosylation of ABI5 targets proteasomal degradation through CUL4-based and KEG ligases and acts as a regulatory switch for seed germination in *Arabidopsis*. Thus, *S*-nitrosylation offers new insights into the regulation of protein stability through CULLIN-related degradation pathways in plants.

The findings by Gibbs *et al*.^7^ and those reported here demonstrate that NO targets ABI5 at both the transcriptional level (through NO-mediated degradation of ERFVIIs) and the posttranslational level (through NO-mediated degradation of ABI5), respectively, highlighting the fact that ABI5 is depleted via a dual NO-responsive mechanism. These two independent mechanisms converging on ABI5 may have evolved to ensure that NO irrevocably removes ABI5 from the seed to promote germination. Several previous reports emphasize that ABI5 protein stability is regulated tightly by multiple different mechanisms^21^,^22^,^23^,^24^. Thus, it is reasonable to speculate that *S*-nitrosylation-mediated ABI5 protein degradation plays an important role in the NO function to regulate seed germination, rather than only through transcriptional regulation^7^. In addition, it is likely to be that other mechanisms of NO-induced ABI5 degradation independently of C153 *S*-nitrosylation may be involved, probably nitrosylating different regulators for ABI5 degradation.

The identification of ABI5 as a direct NO target involved in NO-mediated effects in plant growth and development contributes to our understanding of NO role in plant signal transduction networks and establishes a molecular framework for the NO function during seed germination.

## Methods

### Plant materials and treatments

*Arabidopsis thaliana* ecotype Columbia-0 (Col-0) was the genetic background for all wild-type plants used in this work. Seed stocks of *abi5*, *atnoa1*, *nia1;nia2* and *keg4* mutants were obtained from *Arabidopsis* Biological Resource Center. The *atnoa1*;*nia1;nia2* mutants^11^ and *35S:AHb1* (H3, H7) and *35S:antiAHb1* (L1, L3) lines^27^ were kind gifts from Dr José León (IBMCP-CSIC, Valencia, Spain) and Dr Massimo Delledonne (University of Verona, Verona, Italy), respectively. Dr Xing Wang Deng (Yale University, Connecticut, USA) kindly provided *cul4cs* and *35S:FLAG-CUL4* lines^20^. DEX:*:KEG-HA* lines were previously described^28^.

*Arabidopsis* plants were grown in a growth chamber or greenhouse under 50–60% humidity, a temperature of 22 °C and with a 16-h light/8-h dark photoperiod at 80–100 μE m^−2^ s^−1^ in pots containing a 1:3 vermiculite/soil mixture.

For *in vitro* culture, *Arabidopsis* seeds were surface sterilized in 75% (v/v) sodium hypochlorite and 0.01% (v/v) Triton X-100 for 5 min, and washed three times in sterile water before sowing. Seeds were stratified for 3 days at 4 °C and then sowed on Murashige and Skoog (MS)^29^ solid medium with 2% (w/v) Suc and 0.6% (w/v) agar, and the pH was adjusted to 5.7 with KOH before autoclaving. Seeds were sown in different treatments and plates were sealed and incubated in a controlled environment growth chamber. For NO treatments, *Arabidopsis* seeds were sown in MS medium supplemented with 300 μM NO donors GSNO and SNAP or 300 μM 2-(4-carboxyphenyl)-4,4,5,5-tetramethylimidazoline-1-oxyl-3-oxide (cPTIO) NO scavenger. MG132 proteasome inhibitor (100 μM) and 0.1–5 μM of ABA were used. Whenever required, seeds were transferred every 24 h to a new medium containing fresh compounds. At the end of the treatment, tissues were collected and frozen (−80 °C).

NO donors (SNAP and GSNO), NO scavenger (cPTIO), proteasome inhibitor (MG132), cycloheximide and ABA were purchased from Sigma.

### Mutant screening

M2 seeds of ethyl methane sulfonate-mutagenized Columbia, ethyl methane sulfonate-mutagenized Landsberg *erecta* and fast neutron-mutagenized Columbia were purchased from Lehle Seeds (Round Rock, TX) and each of ∼20,000 M2 seeds (20 batches of ∼1,000 M2 seeds harvested from ∼1,000 M1 seeds) was used for screening to isolate ABA- and cPTIO-insensitive mutants. Screening conditions were described previously^30^, except for (+)-*S*-ABA and cPTIO were used. Briefly, M2 seeds were surface sterilized, sown on 0.8% agar plates supplemented with half strength of MS and 3 μM (+)-S-ABA or 100 μM cPTIO. Seeds were stratified for 4 days and incubated in the presence of ABA for 4 days at room temperature under continuous light condition. Germination in cPTIO was tested under continuous light conditions at room temperature without stratification. Seedlings were transferred to hormone-free media, incubated for several days and transfered to pots for seed harvest.

### Germination assays

To measure ABA, cPTIO, NaCl and mannitol sensitivity, seeds were plated on solid medium composed of MS basal salts, 2% (w/v) Suc or 0.8% w/v agar plates (adjusted to pH 5.8 by 2-(N-Morpholino)ethanesulfonic acid buffer) and different concentrations of ABA (0.1–1 μM), cPTIO (100 μM), SNAP (300 μM), NaCl (100 mM) and mannitol (250 mM). Seed lots to be compared were harvested on the same day from individual plants grown under identical environmental conditions. Each value represents the average germination percentage of 50–100 seeds with the s.e. of three replicates. Experiments were repeated at least three times. For the dormancy assay, seed lots to be compared were freshly harvested on the same day from individual plants grown under identical environmental conditions and sown immediately without stratification. The after-ripening status of dry seed lots was determined following storage for 1–4 weeks. The percentage of seeds with an emerged radicle (germination) or seedlings that germinated and developed green, fully expanded cotyledons was determined every day during 10 days after sowing.

### Detection of endogenous NO

One- to 3-day-old seeds were incubated in a 500-μl solution containing 5 μM of DAF-2DA (Sigma) in buffer Tris–HCl 10 mM, pH 7.4, during 1 h at 25 °C in the dark. Seedlings were then washed three times for 15 min with fresh buffer Tris–HCl 10 mM, pH 7.4. Finally, fluorescence emitted by DAF-2DA was detected on a Leica magnifying glass by excitation at 495 nm and emission at 515 nm. NO depletion was also achieved by adding the scavenger cPTIO (1 mM) to the solution.

### GUS histochemical staining

GUS staining of *ABI5*_*pro*_*:ABI5-GUS* seeds and seedlings was performed as previously described^31^ using 50 mM potassium phosphate buffer (pH 7.0) containing 0.05% (v/v) Triton X-100, 1 mM K_3_Fe(CN)_6_, 1 mM K_4_Fe(CN)_6_, 0.05 M EDTA and 1 mg ml^−1^ X-Gluc (Duchefa). Twenty-four-hour *Arabidopsis* germinated seeds in MS medium as well as endosperms from imbibed seeds with embryo removed after GSNO (1 mM), cPTIO (1 mM) and control (MetOH) treatment were used. Staining was examined after 2 h incubation at 37 °C for germinated seeds and 48 h for endosperms. Samples were mounted in 50% glycerol for imaging.

### Quantitative reverse transcriptase–PCR analysis

Total RNA for quantitative reverse transcriptase–PCR (qRT–PCR) was extracted from 1- to 3-day-old *Arabidopsis* seeds treated with GSNO (300 μM), SNAP (300 μM), cPTIO (300 μM), ABA (5 μM), cycloheximide (1 mM), MG132 (100 μM) and control condition as previously described^32^. Briefly, frozen tissue was powdered with mortar and pestle, and incubated on ice with 550 μl of extraction buffer (0.4 M LiCl, 0.2 M Tris, pH 8, 25 mM EDTA, 1% SDS) and 550 μl chloroform followed by centrifugation for 3 min at 15,800*g* at 4 °C. Next, 500 μl of water-saturated acidic phenol and 200 μl of chloroform were added to the supernatant. After centrifugation for 3 min, 1/3 volume of 8 M LiCl was used for precipitation at −20 °C for 1 h before centrifugation again for 30 min at 4 °C. Pellet was dissolved in 26 μl Diethyl pyrocarbonate treated water and incubated with 10 units of RNase-free DNase I (Roche) 30 min at 37 °C. RNA was precipitated in 3 M NaAc, pH 5.2, and ethanol, washed with 70% ethanol and resuspended in 20 μl Diethyl pyrocarbonate treated water. For 7-day-old *abi5-1;35S:cMyc:ABI5*, *abi5-1;35S:cMyc:ABI5C153S*, *Col-0;35S:cMyc:ABI5* and *Col-0;35S:cMyc:ABI5C153S Arabidopsis* seedlings, total RNA was extracted using TRIzol Reagent as directed by the manufacturer (Invitrogen) and complementary DNA was synthesized using SuperScript Kit (Roche). qRT–PCRs were performed as described previously^15^ in ABI PRISM 7000 Sequence Detection System (Applied Biosystems, Framingham, MA, USA). Amplification was carried out with ‘Brilliant SYBR Green QPCR MasterMix' (Stratagene) according to the manufacturer's instructions. The thermal profile for SYBR Green real-time PCR was 50 °C for 2 min, 95 °C for 10 min, followed by 40 cycles of 95 °C for 15 s and 60 °C for 1 min. To generate the standard curves, cDNA isolated from *Arabidopsis* seedlings was serially diluted 10 × and aliquots of the dilutions were used in standard real-time PCRs. Each value determination was repeated three times, to ensure the slope of the standard curves and to determine the s.d. The concentration of unknown samples was calculated with the ABI-Prism 7000 SDS software, which created threshold cycle values (*C*_t_) and extrapolated relative levels of PCR product from the standard curve. Primers used were *ABI5* (forward 5′-AACATGCATTGGCGGCGGAGT-3′, reverse 5′-TTGTGCCCTTGACTTCAAACT-3′) and *ACTIN8* (forward 5′-AGTGGTCGTACAACCGGTATTGT-3′, reverse 5′-GAGGATAGCATGTGGAAGTGAGAA-3′) as a control.

### Production of recombinant proteins and polyclonal antibodies

Wild-type *ABI5* (kindly provided by Dr Luis Lopez-Molina, University of Geneva, Geneva, Switzerland) and mutated *ABI5C153S* cDNAs were recombined into pET-28a^+^ vector, to obtain fusion proteins with amino-terminal His-tag. Recombinant proteins were expressed in *Escherichia coli* and purified using His-Select Nickel Affinity Gel (IMAC) (Sigma) according to the manufacturer's protocols (Biomedal).

A primary dose of 750 μg purified recombinant protein 6 × His-ABI5 was emulsified in Freunds complete adjuvant (Sigma) and administered subcutaneously in two rabbits. Three doses of the protein (375 μg) emulsified in Freunds incomplete adjuvant (Sigma) were administered at intervals (21 days). After the third booster injection (10 days), blood was collected from the rabbits and the serums were separated. Antibodies (anti-ABI5) were isolated by column chromatography with a protein-G column (GE Healthcare Life Sciences).

### Western blotting

Total protein for western blot analysis was extracted from dormant seeds of Col-0, *abi5-1* and after-ripened seeds of Col-0, *abi5-1* and transgenic lines untreated or treated during 72 h with ABA, cPTIO, GSNO, SNAP, GSNO and SNAP plus MG132 or the proteasome inhibitor cocktail including MG115 (1 μM), MG132 (100 μM) and epoxomicin (0.75 μM). For cycloheximide treatments, transgenic plants were germinated and grown on MS liquid medium during 7 days and cycloheximide (1 mM) alone or in combination with GSNO (500 μM) was added to the medium, and samples were taken at indicated intervals. Tissue was powdered using mortar and pestle, and incubated for 10 min on ice with extraction buffer (50 mM Tris–HCl, pH 7.5, 75 mM NaCl, 15 mM EGTA, 15 mM MgCl_2_, 1 mM DTT, 0.1% Tween 20, 1 mM NaF, 0.2 M NaV, 2 mM Na-pyrophosphate, 60 mM β-glycerolphosphate and 1 × proteases inhibitor mix, Roche) followed by centrifugation for 10 min at 15,800*g* at 4 °C. Protein concentration was determined by the Bio-Rad Protein Assay (Bio-Rad) based on the Bradford method^33^. Sixty micrograms of total protein was loaded per well in SDS-acrylamide/bisacrylamide gel electrophoresis using Tris–glycine–SDS buffer. Proteins were electrophoretically transferred to an Inmobilon-P polyvinylidene difluoride membrane (Millipore) using the Trans-Blot Turbo (Bio-Rad). Membranes were blocked in Tris buffered saline-0,1% Tween 20 containing 5% Blocking Agent and probed with antibodies diluted in blocking buffer. Anti-ABI5 Purified Rabbit Immunoglobulin (Biomedal, 1:5,000), anti-Actin clone 10-B3 Purified Mouse Immunoglobulin (Sigma A0480, 1:20,000), monoclonal anti-polyHistidine−Peroxidase produced in mouse (Sigma A7058, 1:2,000) and ECL-Peroxidase-labelled anti-rabbit (Amersham NA934, 1:20,000) and anti-mouse (Amersham NA931, 1:20,000) antibodies were used in the western blot analyses. Detection was performed using ECL Advance Western Blotting Detection Kit (Amersham) and the chemiluminescence was detected using an Intelligent Dark-Box II, LAS-1000 scanning system (Fujifilm). Quantification of band intensity was performed with ImageJ software. Full-sized uncropped immunoblots of cropped blottings used in figures are included in Supplementary Fig. 12.

### Site-directed mutagenesis

Site-directed mutagenesis of ABI5 was performed using the QuickChange II Site-Directed Mutagenesis Kits (Stratagene Corporate). Plasmid pET28a-*ABI5* was used as template and primers were designed using the tools from Stratagene and synthesized by Isogen. The primers were as follows: forward primer: 5′-CACTTCCAGCTCCGCTTAGTAGGAAGACTGTTGAT-3′and reverse primer 5′-ATCAACAGTCTTCCTACTAAGCGGAGCTGGAAGTG-3′. Mutations were confirmed by sequencing.

### *In vivo* and *in vitro S*-nitrosylation assays

We used the biotin switch method^18^ that converts –SNO into biotinylated groups, to detect *S*-nitrosylated proteins in *Arabidopsis* seed extracts and recombinant purified ABI5 and ABI5C153S, with slight modification.

For *in vitro S*-nitrosylation, purified ABI5 recombinant protein was pre-treated with NO donors SNAP and GSNO (200 μM, Calbiochem), and the glutathionylating agent glutathione (200 μM, Sigma) in the dark at room temperature for 30 min with regular vortexing. Treatment with the reducing agent (DTT, 20 mM; Sigma) after GSNO incubation was carried out for 1 h under the same conditions, to check reversibility of the modification. Reagents were removed by precipitation with two volumes of cold acetone and proteins were assayed by the biotin switch method.

*In vivo S*-nitrosylation of ABI5 was carried out with *Arabidopsis* dry seeds homogenized in extraction buffer (50 mM Tris–HCl, pH 7.5, 75 mM NaCl, 15 mM EGTA, 15 mM MgCl_2_, 0.1% Tween 20, 1 mM NaF, 0.2 M NaV, 2 mM Na-pyrophosphate, 60 mM β-glycerolphosphate) containing Complete Protease Inhibitor Cocktail (Roche). Seeds extracts (1 mg) were incubated with GSNO (1 mM) and proteasome inhibitor MG132 (100 μM) in the dark for 30 min with repeated vortexing. Samples treated with DTT (20 mM) were also kept for 1 h under the same conditions and were used as a negative control. Proteins were recovered by precipitation with two volumes of acetone for 20 min at −20 °C, to remove excess of GSNO/DTT and assayed by the biotin switch method.

For the biotin switch, extracts or recombinant proteins were incubated with 20 mM *S*-methyl-methanethiosulfonate and 2.5% SDS at 50 °C for 30 min with frequent vortexing, to block free Cys. *S*-methyl-methanethiosulfonate was removed by protein precipitation with two volumes of cold acetone and proteins were disolved in 0.1 ml of RB buffer (25 mM Hepes, 1 mM EDTA and 1% SDS, pH 7.7) per mg of protein. After addition of 1 mM HPDP biotin (Pierce, Rockford, IL) and 1 mM ascorbic acid, the mixture was incubated for 1 h at room temperature in the dark with intermittent vortexing. *In vivo* biotinylated proteins were purified by immunoprecipitation with neutravidin or an IPA (protein A/G Ultralink Resin, Pierce) anti-biotin antibody, overnight at 4 °C with 15 μl IPA per mg of protein, preincubated with 2 μl of anti-biotin antibody (Sigma B7653, 1:2,000) as described elsewhere^34^. Briefly, beads were washed three times with HEN buffer (100 mM Hepes, 1 mM EDTA and 0.1 mM neocuproine, pH 7.8) and bound proteins were eluted with 10 mM DTT in SDS–PAGE solubilization buffer, loaded in 10% SDS–PAGE and transferred to a polyvinylidene difluoride membrane, to detect ABI5 with anti-ABI5 Purified Rabbit Immunoglobulin (Biomedal, 1:5,000). *In-vitro* biotinylated proteins with SDS–PAGE solubilization buffer were loaded in 12% SDS–PAGE, visualized by Brilliant Blue-G Colloidal Stain (Sigma) and protein bands were analysed by matrix-assisted laser desorption/ionization–tandem time of flight (MALDI–TOF/TOF). Alternatively, purified proteins were analysed directly by nano-liquid chromatography (LC) and ion-trap tandem mass spectrometry (MS/MS).

### Mass spectrometry

For tryptic digestion in reducing conditions, Coomassie-stained bands were excised manually, deposited in 96-well plates and processed automatically in a Proteineer DP (Bruker Daltonics, Bremen, Germany). The digestion protocol^35^ was used with minor modifications. Gel plugs were washed first with 50 mM ammonium bicarbonate and second with acetonitrile (ACN) before reduction with 10 mM DTT in 25 mM ammonium bicarbonate solution, and alkylation was carried out with 55 mM iodoacetamide in 50 mM ammonium bicarbonate solution. Gel pieces were then rinsed with 50 mM ammonium bicarbonate and with ACN, and then dried under a stream of nitrogen. Modified porcine trypsin (sequencing grade; Promega, Madison, WI) was added at a final concentration of 16 ng μl^−1^ in 25% ACN/50 mM ammonium bicarbonate solution and the digestion was led to proceed at 37 °C for 6 h. The reaction was stopped by adding 0.5% trifluoroacetic acid for peptide extraction. Digestion in non-reducing conditions was done essentially with the same protocol, but no DTT and iodoacetamide were used. In both cases, tryptic peptides were dried by speed-vacuum centrifugation and resuspended in 4 μl of MALDI solution (30% ACN+15% isopropanol+0.1% trifluoroacetic acid). Twenty per cent of each peptide mixture was deposited onto a 386-well OptiTOF Plate (Applied Biosystems) and allowed to dry at room temperature. A 0.8-μl aliquot of matrix solution (3 mg ml^−1^ CHCA in MALDI solution) was then added onto the dried digests and allowed to dry at room temperature.

For MALDI peptide mass fingerprinting (PMF) and MS/MS analysis, samples were automatically processed in an ABi 4800 MALDI–TOF/TOF mass spectrometer (Applied Biosystems) in positive ion reflector mode (ion acceleration voltage was 25 kV for MS acquisition and 1 kV for MS/MS) and the spectra were stored into the ABi 4000 Series Explorer Spot Set Manager. PMF and MS/MS fragment ion spectra were smoothed and corrected to zero baseline using routines embedded in ABi 4000 Series Explorer Software v3.6. Each PMF spectrum was internally calibrated with the mass signals of trypsin autolysis ions to reach a typical mass measurement accuracy of <25 p.p.m. Known trypsin and keratin mass signals, as well as potential sodium and potassium adducts (+21 Da and +39 Da) were removed from the peak list.

To submit the combined PMF and MS/MS data to MASCOT software v.2.1 (Matrix Science, London, UK), GPS Explorer v4.9 was used, searching in the non-redundant NCBI protein database (Viridiplantae) with peptide mass tolerance of 75 p.p.m. for MALDI–TOF/TOF analysis.

The remaining 80% of the samples were analysed by LC coupled to electrospray ion-trap mass spectrometry MS/MS using Ultimate 3000 nano LC (Dionex, Amsterdam, The Netherland) and a 75-mm I.D., 100 mm reverse-phase column, at 300 nl min^−1^ flow, coupled to a Bruker HCT Ultra ion-trap mass spectrometer (Bruker Daltonics, Bremen, Germany) working in dynamic exclusion mode. For protein identification, LC coupled to electrospray ion-trap mass spectrometry MS/MS spectra were transferred to BioTools 2.0 interface (Bruker Daltonics), to search in the NCBInr database using a licensed version of Mascot v.2.2.04 search engine (www.matrixscience.com; Matrix Science). Search parameters were set as follows: in reduced samples, carbamidomethyl cystein was set as fixed modification by the treatment with iodoacetamide, oxidized methionines as variable modification, peptide mass tolerance of 0.5 Da for the parental mass and fragment masses, and one missed cleavage site. In the case of non-reduced samples, biotin-HPDP cysteine modification was set as variable modification.

### Yeast two-hybrid assay

*ABI5* and *ABI5C153S* were cloned in the pDEST22 and pDEST32 vectors using the GATEWAY technology. Prey and bait clones were grown for 3 days on DOB-W and DOB-L (MP Biomedicals) plates from their corresponding frozen stocks. YPAD medium was inoculated in parallel with bait and prey cells, and incubated overnight at 28 °C with shaking (200 r.p.m.). After overnight incubation, the bait culture was added to prey and mating was allowed 48 h by incubating at 28 °C without shaking. Settled cells were resuspended and used to inoculate plates containing diploid selection media (DOB-L-W). After 1 day of growth at 28 °C and vigorous shaking, diploid cells were resuspended and spotted onto diploid selection and screening (DOB-L-W-Histidine±3-Amino-1,2,4-triazole; 3-AT, Sigma) plates. Positive colonies were visible after 2–5 days of growth at 28 °C.

### Electrophoretic mobility shift assay

For the electrophoretic mobility shift assay, 10 ng of a double-stranded biotinylated probe 5′-GATCCTCTCGCGTACAATAAAGTCAGACACGTGGCATGTCACCAACGTAGCGTATGCGTA-3′(ABRE-binding site motif underlined)^36^ was incubated with 1.5 μg wild-type ABI5 or mutant ABI5C153S recombinant protein in the presence and absence of GSNO (1 mM) and DTT (1 mM) as described previously^37^. Basically, DNA-binding reactions were performed in a buffer containing 10 mM Tris, pH 8, 1 mM EDTA, 100 mM NaCl, 2 mM DTT and 10% glycerol. Protein extracts were mixed with labelled DNA, 0.5 μg poly(dI-dC) and BSA (250 μg μl^−1^) and incubated for 30 min on ice. Electrophoretic mobility shift assay to separate free and bound DNA was in 6% polyacrylamide gel (40:1 bisacrylamide cast in 0.5 × TBE; TBE is 89 mM Tris, 89 mM boric acid and 2 mM EDTA).

### Co-immunoprecipitation and pull-down assays

For haemagglutinin (HA) and FLAG pull-down assays, the proteins were extracted with lysis buffer (100 mM Tris–HCl, pH 7.5, 150 mM NaCl, 0.1% Tween-20) supplemented with 1 mM phenylmethanesulfonyl fluoride and protease inhibitors (Roche). Extracts were cleared by centrifugation and protein concentration was determined by Bradford assay. One milligram of soluble proteins was treated with GSNO (1 mM) in darkness at room temperature during 1 h. After treatment, extracts were immunoprecipitated using anti-HA Affinity Matrix (Roche) and anti-FLAG M2 Matrix (Sigma) for ABI5/KEG and ABI5/CUL4 interactions, respectively. Extracts and beads were incubated during 2 h at 4 °C and beads were washed two times in lysis buffer. Next, the beads were incubated at 4 °C during 2 h with 1 mg of ABI5 and ABI5C153S soluble proteins previously treated with GSNO (1 mM) in darkness at room temperature during 1 h. After incubation, beads were washed three times in lysis buffer and proteins were eluted from beads in SDS sample buffer. The proteins were visualized using anti-MYC antibodies (Abcam ab62928, 1:10,000), anti-HA antibodies (Roche 12013819001, 1:2,000) and anti-FLAG antibodies (Sigma F1804, 1:1,000).

### Generation of transgenic *Arabidopsis* plants

*ABI5* and *ABI5C153S* were cloned in the pEarleyGate 203 vector^38^ using the GATEWAY technology and the following primers (ABI5-F 5′-ATGGTAACTAGAGAAACGAAGTTGACG-3′; ABI5-R 5′-TTAGAGTGGACAACTCGGG-3′). Similarly, *ABI5*_*pro*_*:ABI5* was cloned in the binary pGWB3 vector^39^ fused to *GUS*. The constructs generated (Supplementary Fig. 11a) were used to transform the C58C1 (pGV2260) *Agrobacterium* strain^40^. For plant transformation, *Arabidopsis* plants (Col-0, *abi5-1* or *abi5-7*) were transformed by the floral dip method^41^ as described previuosly^42^. Seeds were harvested and plated on selection medium to identify T1 transgenic plants. Approximately 100 of the T2 seeds were plated on selection medium MS agar plates and transgenic lines with a 3:1 (resistant/sensitive) segregation ratio were selected. T3 progenies homozygous for selection medium resistance were used for further studies.

## Additional information

**How to cite this article:** Albertos, P. *et al*. *S*-nitrosylation triggers ABI5 degradation to promote seed germination and seedling growth. *Nat. Commun.* 6:8669 doi: 10.1038/ncomms9669 (2015).

## Supplementary Material

Supplementary Information Supplementary Figures 1-12 and Supplementary References
