# Supplementary material for: S-nitrosylation triggers ABI5 degradation to promote seed germination and seedling growth
Source: Nat Commun. 2015 Oct 23;6:8669. doi: 10.1038/ncomms9669 (PMC4639896; doi:10.1038/ncomms9669)
Supplement: Supplementary Information — Supplementary Figures 1-12 and Supplementary References [file ncomms9669-s1.pdf]

Supplementary Information

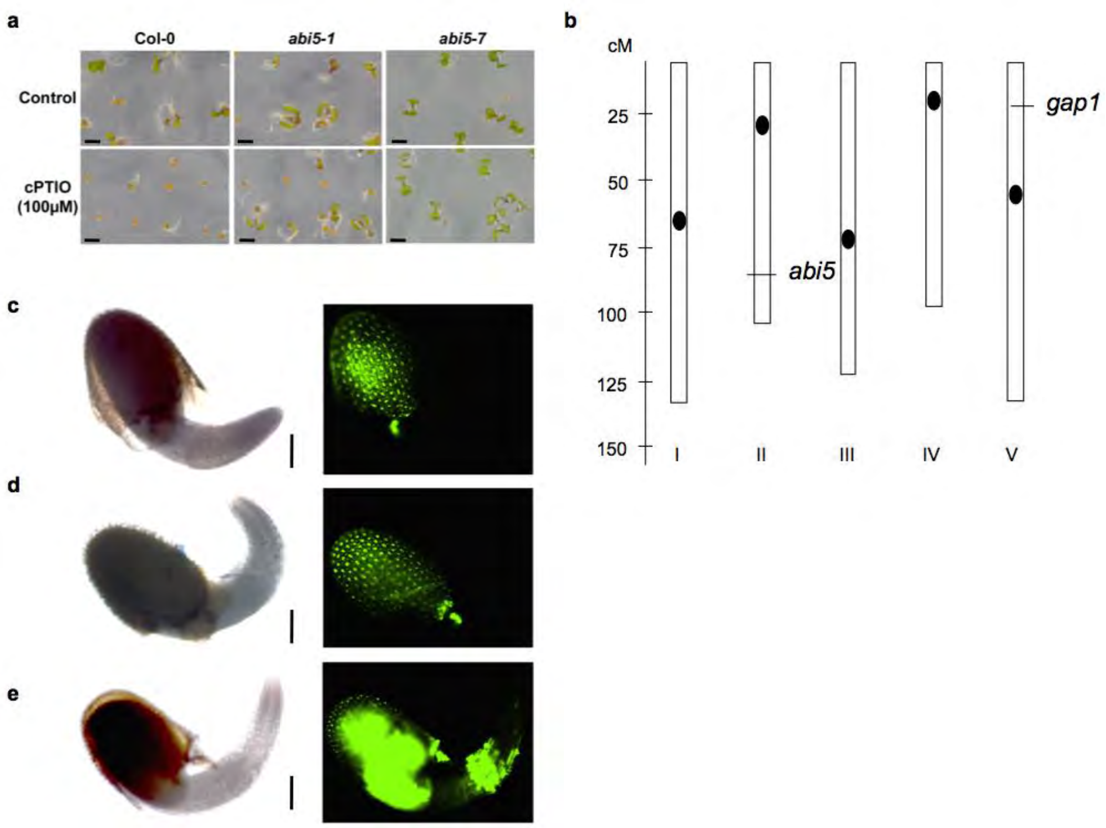

Supplementary Figure 1. Identification and characterization of *gap* mutants. Controls of endogenous NO detection in seeds by using DAF-2DA.

a, Post-germinative growth of wild-type (Col-0), *abi5-1* and *abi5-7* seedlings in media containing 0 and 100 µM cPTIO after 6 days. Scale bars 1 mm.

b, Chromosomal position of the two identified loci (*abi5, gap1*). cM, centimorgan.

c, Autofluorescence of the testa tissues in untreated control seeds. Scale bar 100 µm.

d, Fluorescence of seeds treated with the NO scavenger cPTIO (1 mM) that were stratified during 3 days at 4ºC and germinated for 2 days at 21ºC on agar plates and then subjected to DAF-2DA incubation. Scale bar 100 µm.

e, NO production detected by DAF-2DA as in Figure 1c. For detection of endogenous NO production seeds were stratified for 3 days at 4ºC and grown for 1 to 3 days at 21ºC on agar plates and then subjected to DAF-2DA incubation. Scale bar 100 µm.

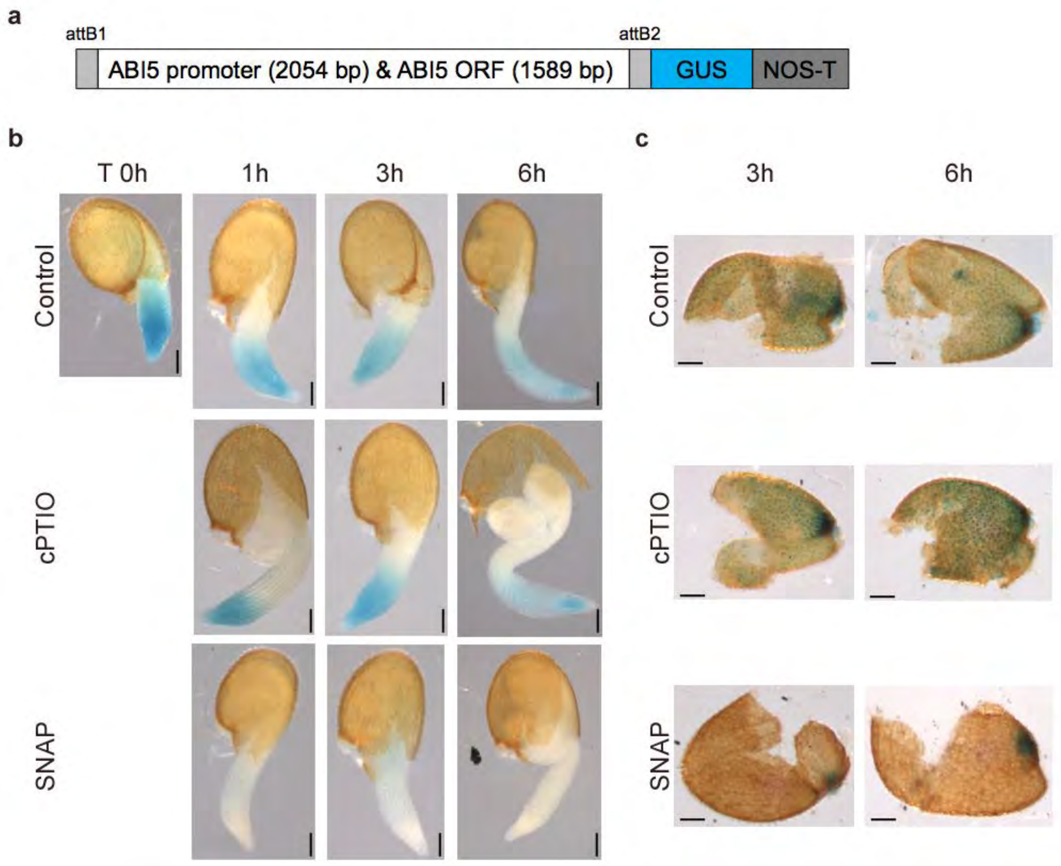

Supplementary Figure 2. Tissue specific reduction of ABI5-GUS protein.

a, Construct used to generate the *pABI5:ABI5-GUS* transgenic lines.

b, Representative GUS staining images of *pABI5:ABI5-GUS* 1 to 2-day-old seeds germinated on MS plates. Scale bars 100 µm.

c, Representative GUS staining images of *pABI5:ABI5-GUS* 15-minutes imbibed testas after embryo removal. Scale bars 100 µm.

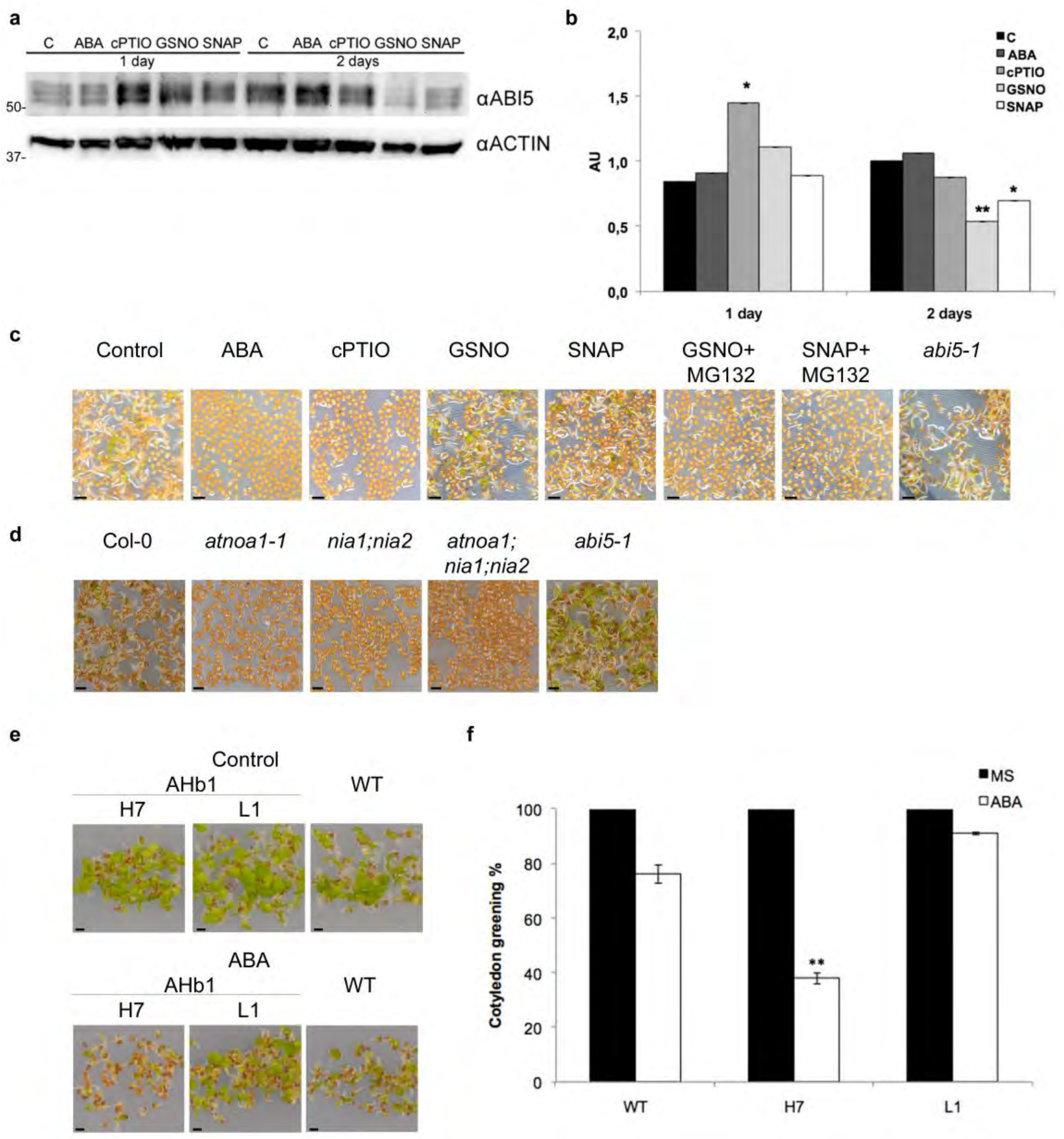

Supplementary Figure 3. NO-promoted ABI5 degradation and seed germination phenotypes.

a, GSNO and SNAP treatments promote ABI5 degradation in 2 days ABA (5 µM)-treated after ripened seeds. Immunoblot analysis of ABI5 protein levels in seed extracts of Col-0, treated with or without (C) ABA, NO scavenger (cPTIO) and NO donors (GSNO and SNAP). Actin protein levels are shown as a loading control.

b, Quantitative data of immunoblot analysis of ABI5 protein levels in seed extracts of Col-0, treated with or without (C) ABA, NO scavenger (cPTIO) and NO donors (GSNO and SNAP). Values represent the mean ±SE (*n*=3) and normalized against actin. Asterisks indicate significant differences compared with C (*t*-test, **P*<0.05, ***P*<0.01). AU, arbitrary units.

c, Representative seed germination images of wild-type (Col-0) dormant seeds grown for 4 days on MS agar plates untreated (Control, C), or supplemented with 5 µM ABA, 300 µM of the NO-scavenger cPTIO, 300 µM of the NO-donors GSNO and SNAP and 300 µM GSNO or SNAP plus 100 µM of the proteasome inhibitor MG132. Phenotype of *abi5-1* mutant is included as a control. Scale bars 1 mm.

d, Representative seed germination images of wild-type (Col-0) and mutants *atnoa1-1, nia1nia2, atnoa1-2nia1nia2,* and *abi5-1* 4-day-old seedlings in the presence of 0.1µM ABA highlighting germination inhibition. Scale bars 1 mm.

e, Representative seed germination images of WT, *35S:AHb1* (H7) and *35S:antiAHb1* (L1) 4-day-old seedlings in MS (Control) and 0.1 µM ABA. Scale bars 1 mm.

f, Graph showing the quantification of seed germination in WT, *35S:AHb1* (H7) and *35S:antiAHb1* (L1) 4-day-old seedlings in MS (Control) and 0.1 µM ABA. Values represent the mean ±SE (*n*=3). Asterisks indicate significant differences compared with WT (*t*-test, ***P*<0.01).

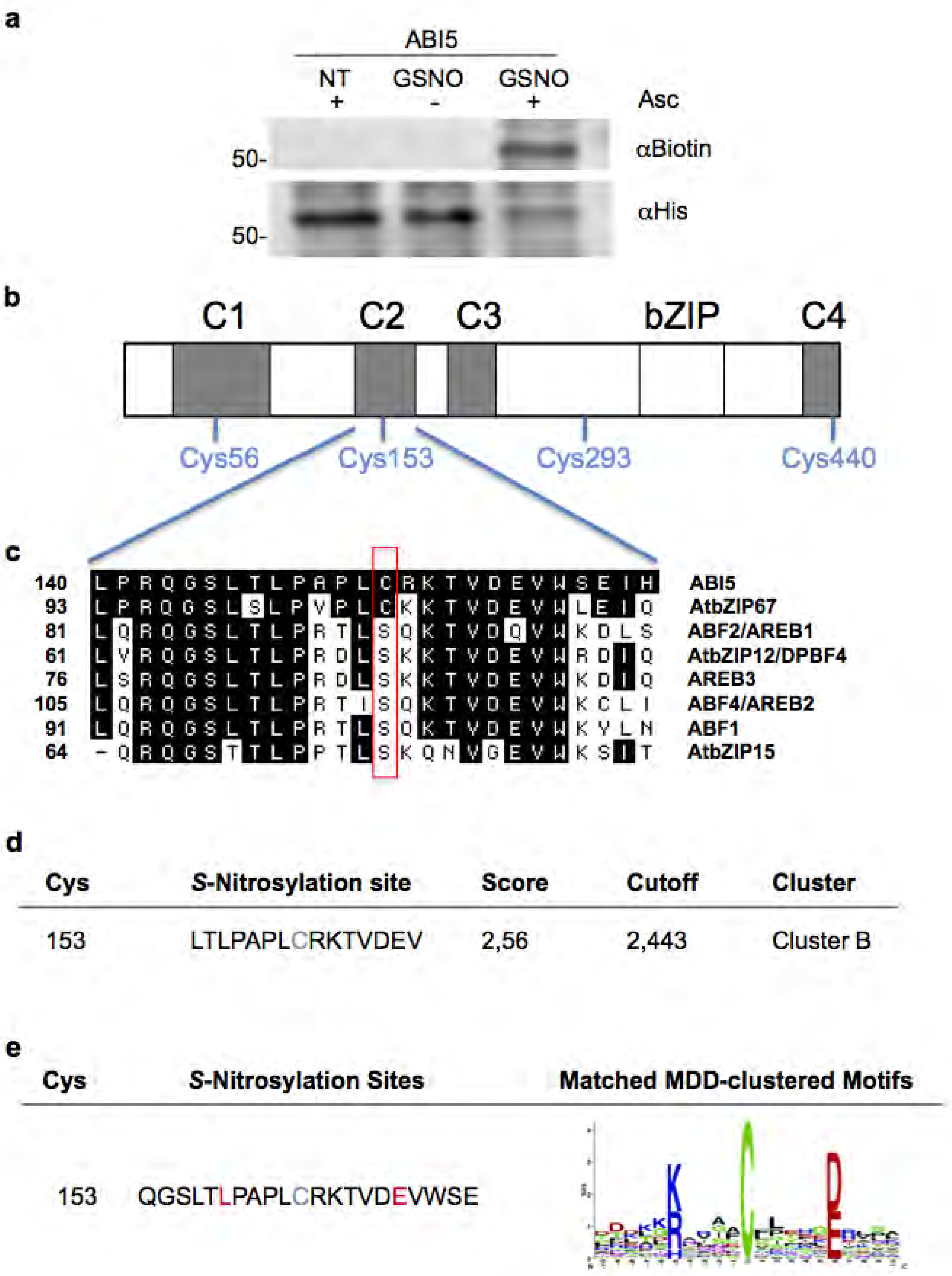

Supplementary Figure 4. ***In vitro S*-nitrosylation of ABI5 and identification of the candidate ABI5 Cys residue *in silico*.**

a, *In vitro S*-nitrosylation of wild-type ABI5 recombinant protein by the NO donor GSNO (200 µM). Sodium ascorbate (Asc) was used to specifically detect S-nitrosylated SNO-ABI5. ABI5 protein loading was detected by anti-His antibody.

b, Schematic ABI5 structure showing the conserved domains (C1-4 and bZIP) and the position of the 4 Cys residues.

c, Sequence alignment of ABI5 (At2g36270) protein with other members of the ABI5-like bZIP family in Arabidopsis. Positions with identical amino acids residues are highlighted in black. The Cys residue is red boxed.

d, Computational prediction of ABI5 *S*-nitrosylation sites. GPS-SNO (http://sno.biocuckoo.org)[1] has been used to calculate ABI5 Cys targets of *S*-nitrosylation using the High Threshold option.

e, dbSNO database (http://csb.cse.yzu.edu.tw/SNOSite/index.html)[2] has been also used to predict ABI5 S-nitrosylation sites identifying the matched MDD-clustered motifs of the target cysteines. MDD: maximal dependence decomposition. Target Cys of the MDD-clustered motif is in green, and the maximal dependence with the occurrence of basic (blue) or acid (red) amino acids in each position is marked. Target Cys of the *S*-nitrosylation sites are in blue and amino acids that match with the respective MDD-clustered motif are in red.

Mascot Search Results

Protein View

Match to: **gi|18404091** Score: **682**
protein abscisic acid-insensitive 5 [Arabidopsis thaliana]
Found in search of MCR3_sin reducir_30%_RC6_01_5646.mgf

Nominal mass (M_r): **46978**; Calculated pI value: **9.08**
NCBI BLAST search of gi|18404091 against nr
Unformatted sequence string for pasting into other applications

Taxonomy: Arabidopsis thaliana
Links to retrieve other entries containing this sequence from NCBI Entrez:
gi|75313515 from Arabidopsis thaliana
gi|4510349 from Arabidopsis thaliana
gi|13346151 from Arabidopsis thaliana
gi|111074502 from Arabidopsis thaliana
gi|330254132 from Arabidopsis thaliana

Variable modifications: Oxidation (M),Biotin-HPDP (C)
Cleavage by Trypsin: cuts C-term side of KR unless next residue is P
Sequence Coverage: **25%**

Matched peptides shown in **Bold Red**

1 MVTRETK**LTS EREVESSMAQ ARHNGGGGGE NHPFTSLGR**Q SSIYSLTLDE
51 FQHALCENGK NFGSMNMDEF LVSIWNAEEN NNNQQQAAAA AGSHSVPANH
101 NGFNNNNNNG GEGGVGVFSG GSRGNEDANN K**RGIANESSL PRQGSLTLPA**
151 **PLCRKTVDEV WSEIHRGGGS GNGGDSNGRS SSSNGQNNAQ NGGETAARQP**
201 **TFGEMTLEDF LVK**AGVVREH PTNPKPNPNP NQNQNPSSVI PAAAQQQLYG
251 VFQGTGDPSF PGQAMGVGDP SGYAKRTGGG GYQQAPPVQA GVCYGGGVGF
301 GAGGQQMGMV GPLSPVSSDG LGHGQVDNIG GQYGVDMGGL RGRKRVVDGP
351 VEKVVERRQR RMIKNRESAA RSRARKQAYT VELEAELNQL KEENAQLKHA
401 LAELERKRKQ QYFESLKSRA QPKLPKSNGR LRTLMRNPSC PL

Show predicted peptides also

Sort Peptides By ◉ Residue Number ○ Increasing Mass ○ Decreasing Mass

Start - End	Observed	Mr(expt)	Mr(calc)	Delta	Miss	Sequence
8 - 22	855.3200	1708.6254	1708.8050	-0.1795	1	K.LTSEREVESSMAQAR.H Oxidation (M) (Ions score 55)
8 - 22	855.3200	1708.6254	1708.8050	-0.1795	1	K.LTSEREVESSMAQAR.H Oxidation (M) (Ions score 49)
8 - 22	570.5900	1708.7482	1708.8050	-0.0568	1	K.LTSEREVESSMAQAR.H Oxidation (M) (Ions score 10)
8 - 22	570.6900	1709.0482	1708.8050	0.2432	1	K.LTSEREVESSMAQAR.H Oxidation (M) (Ions score 57)
13 - 22	554.2100	1106.4054	1106.5026	-0.0971	0	R.EVESSMAQAR.H (Ions score 73)
23 - 39	564.7700	1691.2882	1692.7717	-1.4835	0	R.HNGGGGGENHPFTSLGR.Q (Ions score 39)
23 - 39	846.8700	1691.7254	1692.7717	-1.0462	0	R.HNGGGGGENHPFTSLGR.Q (Ions score 22)
23 - 39	847.2800	1692.5454	1692.7717	-0.2263	0	R.HNGGGGGENHPFTSLGR.Q (Ions score 88)
23 - 39	847.2800	1692.5454	1692.7717	-0.2263	0	R.HNGGGGGENHPFTSLGR.Q (Ions score 125)
23 - 39	847.2800	1692.5454	1692.7717	-0.2263	0	R.HNGGGGGENHPFTSLGR.Q (Ions score 8)
23 - 39	565.2300	1692.6682	1692.7717	-0.1035	0	R.HNGGGGGENHPFTSLGR.Q (Ions score 62)
23 - 39	565.2300	1692.6682	1692.7717	-0.1035	0	R.HNGGGGGENHPFTSLGR.Q (Ions score 64)
23 - 39	565.2700	1692.7882	1692.7717	0.0165	0	R.HNGGGGGENHPFTSLGR.Q (Ions score 54)
23 - 39	565.4600	1693.3582	1692.7717	0.5865	0	R.HNGGGGGENHPFTSLGR.Q (Ions score 46)
23 - 39	847.7600	1693.5054	1692.7717	0.7338	0	R.HNGGGGGENHPFTSLGR.Q (Ions score 64)
23 - 39	847.7800	1693.5454	1692.7717	0.7738	0	R.HNGGGGGENHPFTSLGR.Q (Ions score 62)
23 - 39	565.5500	1693.6282	1692.7717	0.8565	0	R.HNGGGGGENHPFTSLGR.Q (Ions score 61)
23 - 39	565.6100	1693.8082	1692.7717	1.0365	0	R.HNGGGGGENHPFTSLGR.Q (Ions score 60)
23 - 39	565.7400	1694.1982	1692.7717	1.4265	0	R.HNGGGGGENHPFTSLGR.Q (Ions score 55)
132 - 142	600.4100	1198.8054	1198.6418	0.1636	1	K.RGIANESSLPR.Q (Ions score 83)
133 - 142	1043.4200	1042.4127	1042.5407	-0.1280	0	R.GIANESSLPR.Q (Ions score 31)
133 - 142	522.6100	1043.2054	1042.5407	0.6648	0	R.GIANESSLPR.Q (Ions score 43)
143 - 154	842.4900	1682.9654	1682.8670	0.0984	0	R.QGSLTLPAPLCR.K Biotin-HPDP (C) (Ions score 48)
143 - 154	562.0100	1683.0082	1682.8670	0.1412	0	R.QGSLTLPAPLCR.K Biotin-HPDP (C) (Ions score 30)
155 - 166	749.7900	1497.5654	1497.7576	-0.1921	1	R.KTVDEVWSEIHR.G (Ions score 99)
155 - 166	500.2200	1497.6382	1497.7576	-0.1194	1	R.KTVDEVWSEIHR.G (Ions score 54)
155 - 166	749.8500	1497.6854	1497.7576	-0.0721	1	R.KTVDEVWSEIHR.G (Ions score 88)
155 - 166	749.9000	1497.7854	1497.7576	0.0279	1	R.KTVDEVWSEIHR.G (Ions score 88)
155 - 166	500.5200	1498.5382	1497.7576	0.7806	1	R.KTVDEVWSEIHR.G (Ions score 54)
156 - 166	685.7500	1369.4854	1369.6626	-0.1772	0	K.TVDEVWSEIHR.G (Ions score 65)
156 - 166	685.8200	1369.6254	1369.6626	-0.0372	0	K.TVDEVWSEIHR.G (Ions score 67)
156 - 166	685.8200	1369.6254	1369.6626	-0.0372	0	K.TVDEVWSEIHR.G (Ions score 56)
156 - 166	685.8800	1369.7454	1369.6626	0.0828	0	K.TVDEVWSEIHR.G (Ions score 55)
156 - 166	685.8900	1369.7654	1369.6626	0.1028	0	K.TVDEVWSEIHR.G (Ions score 57)
156 - 166	685.9100	1369.8054	1369.6626	0.1428	0	K.TVDEVWSEIHR.G (Ions score 60)
156 - 166	685.9200	1369.8254	1369.6626	0.1628	0	K.TVDEVWSEIHR.G (Ions score 60)
156 - 166	685.9500	1369.8854	1369.6626	0.2228	0	K.TVDEVWSEIHR.G (Ions score 53)
156 - 166	685.9600	1369.9054	1369.6626	0.2428	0	K.TVDEVWSEIHR.G (Ions score 58)
156 - 166	457.7100	1370.1082	1369.6626	0.4455	0	K.TVDEVWSEIHR.G (Ions score 26)
167 - 198	974.9900	2921.9482	2921.2229	0.7253	1	R.GGGSGNGGDSNGRSSSSNGQNNAQNGGETAAR.Q (Ions score 16)
199 - 213	877.3700	1752.7254	1753.8597	-1.1342	0	R.QPTFGEMTLEDFLVK.A (Ions score 33)
199 - 213	877.3700	1752.7254	1753.8597	-1.1342	0	R.QPTFGEMTLEDFLVK.A (Ions score 15)
199 - 213	877.3800	1752.7454	1753.8597	-1.1142	0	R.QPTFGEMTLEDFLVK.A (Ions score 29)
199 - 213	877.3800	1752.7454	1753.8597	-1.1142	0	R.QPTFGEMTLEDFLVK.A (Ions score 17)
199 - 213	877.3900	1752.7654	1753.8597	-1.0942	0	R.QPTFGEMTLEDFLVK.A (Ions score 31)
199 - 213	877.3900	1752.7654	1753.8597	-1.0942	0	R.QPTFGEMTLEDFLVK.A (Ions score 18)
199 - 213	877.3900	1752.7654	1753.8597	-1.0942	0	R.QPTFGEMTLEDFLVK.A (Ions score 32)
199 - 213	877.3900	1752.7654	1753.8597	-1.0942	0	R.QPTFGEMTLEDFLVK.A (Ions score 32)
199 - 213	877.4000	1752.7854	1753.8597	-1.0742	0	R.QPTFGEMTLEDFLVK.A (Ions score 32)
199 - 213	877.4000	1752.7854	1753.8597	-1.0742	0	R.QPTFGEMTLEDFLVK.A (Ions score 17)
199 - 213	877.9000	1753.7854	1753.8597	-0.0742	0	R.QPTFGEMTLEDFLVK.A (Ions score 69)
199 - 213	585.6400	1753.8982	1753.8597	0.0385	0	R.QPTFGEMTLEDFLVK.A (Ions score 51)
199 - 213	885.3800	1768.7454	1769.8546	-1.1092	0	R.QPTFGEMTLEDFLVK.A Oxidation (M) (Ions score 51)
199 - 213	885.8400	1769.6654	1769.8546	-0.1892	0	R.QPTFGEMTLEDFLVK.A Oxidation (M) (Ions score 48)
199 - 213	885.8600	1769.7054	1769.8546	-0.1492	0	R.QPTFGEMTLEDFLVK.A Oxidation (M) (Ions score 53)
199 - 213	885.8700	1769.7254	1769.8546	-0.1292	0	R.QPTFGEMTLEDFLVK.A Oxidation (M) (Ions score 50)
199 - 213	885.8700	1769.7254	1769.8546	-0.1292	0	R.QPTFGEMTLEDFLVK.A Oxidation (M) (Ions score 70)
199 - 213	885.8700	1769.7254	1769.8546	-0.1292	0	R.QPTFGEMTLEDFLVK.A Oxidation (M) (Ions score 54)
199 - 213	885.8800	1769.7454	1769.8546	-0.1092	0	R.QPTFGEMTLEDFLVK.A Oxidation (M) (Ions score 50)
199 - 213	885.8800	1769.7454	1769.8546	-0.1092	0	R.QPTFGEMTLEDFLVK.A Oxidation (M) (Ions score 66)
199 - 213	885.8800	1769.7454	1769.8546	-0.1092	0	R.QPTFGEMTLEDFLVK.A Oxidation (M) (Ions score 51)
199 - 213	885.8800	1769.7454	1769.8546	-0.1092	0	R.QPTFGEMTLEDFLVK.A Oxidation (M) (Ions score 44)
199 - 213	885.8900	1769.7654	1769.8546	-0.0892	0	R.QPTFGEMTLEDFLVK.A Oxidation (M) (Ions score 51)
199 - 213	885.8900	1769.7654	1769.8546	-0.0892	0	R.QPTFGEMTLEDFLVK.A Oxidation (M) (Ions score 66)
199 - 213	885.9000	1769.7854	1769.8546	-0.0692	0	R.QPTFGEMTLEDFLVK.A Oxidation (M) (Ions score 64)
199 - 213	885.9000	1769.7854	1769.8546	-0.0692	0	R.QPTFGEMTLEDFLVK.A Oxidation (M) (Ions score 66)
199 - 213	885.9000	1769.7854	1769.8546	-0.0692	0	R.QPTFGEMTLEDFLVK.A Oxidation (M) (Ions score 61)
199 - 213	885.9000	1769.7854	1769.8546	-0.0692	0	R.QPTFGEMTLEDFLVK.A Oxidation (M) (Ions score 62)
199 - 213	885.9000	1769.7854	1769.8546	-0.0692	0	R.QPTFGEMTLEDFLVK.A Oxidation (M) (Ions score 70)
199 - 213	885.9000	1769.7854	1769.8546	-0.0692	0	R.QPTFGEMTLEDFLVK.A Oxidation (M) (Ions score 67)
199 - 213	885.9000	1769.7854	1769.8546	-0.0692	0	R.QPTFGEMTLEDFLVK.A Oxidation (M) (Ions score 72)
199 - 213	590.9800	1769.9182	1769.8546	0.0636	0	R.QPTFGEMTLEDFLVK.A Oxidation (M) (Ions score 52)
199 - 213	886.0500	1770.0854	1769.8546	0.2308	0	R.QPTFGEMTLEDFLVK.A Oxidation (M) (Ions score 66)

```
LOCUS       NP_565840                442 aa            linear   PLN 28-MAY-2011
DEFINITION  protein abscisic acid-insensitive 5 [Arabidopsis thaliana].
ACCESSION   NP_565840
VERSION     NP_565840.1  GI:18404091
DBSOURCE    REFSEQ: accession NM_129185.3
KEYWORDS    .
SOURCE      Arabidopsis thaliana (thale cress)
  ORGANISM  Arabidopsis thaliana
            Eukaryota; Viridiplantae; Streptophyta; Embryophyta; Tracheophyta;
            Spermatophyta; Magnoliophyta; eudicotyledons; core eudicotyledons;
            rosids; malvids; Brassicales; Brassicaceae; Camelineae;
            Arabidopsis.
REFERENCE   1  (residues 1 to 442)
  AUTHORS   Lin,X., Kaul,S., Rounsley,S., Shea,T.P., Benito,M.I., Town,C.D.,
            Fujii,C.Y., Mason,T., Bowman,C.L., Barnstead,M., Feldblyum,T.V.,
            Buell,C.R., Ketchum,K.A., Lee,J., Ronning,C.M., Koo,H.L.,
            Moffat,K.S., Cronin,L.A., Shen,M., Pai,G., Van Aken,S., Umayam,L.,
            Tallon,L.J., Gill,J.E., Adams,M.D., Carrera,A.J., Creasy,T.H.,
            Goodman,H.M., Somerville,C.R., Copenhaver,G.P., Preuss,D.,
            Nierman,W.C., White,O., Eisen,J.A., Salzberg,S.L., Fraser,C.M. and
            Venter,J.C.
  TITLE     Sequence and analysis of chromosome 2 of the plant Arabidopsis
            thaliana
  JOURNAL   Nature 402 (6763), 761-768 (1999)
   PUBMED   10617197
REFERENCE   2  (residues 1 to 442)
  CONSRTM   Arabidopsis TAIR10 Release
  TITLE     Direct Submission
  JOURNAL   Submitted (18-FEB-2011) Department of Plant Biology, Carnegie
            Institution, 260 Panama Street, Stanford, CA, USA
COMMENT     REVIEWED REFSEQ: This record has been curated by TAIR. The
            reference sequence is identical to AEC09226.
            Method: conceptual translation.
FEATURES             Location/Qualifiers
     source          1..442
                     /organism="Arabidopsis thaliana"
                     /db_xref="taxon:3702"
                     /chromosome="2"
                     /ecotype="Columbia"
     Protein         1..442
                     /product="protein abscisic acid-insensitive 5"
                     /calculated_mol_wt=46876
     Region          356..410
                     /region_name="bZIP_1"
                     /note="bZIP transcription factor; cl02576"
                     /db_xref="CDD:198728"
     CDS             1..442
                     /gene="ABI5"
                     /locus_tag="AT2G36270"
                     /gene_synonym="ABA INSENSITIVE 5; ABI5; F2H17.12;
                     F2H17_12; GIA1; GROWTH-INSENSITIVITY TO ABA 1"
                     /coded_by="NM_129185.3:176..1504"
                     /inference="Similar to DNA
                     sequence:INSD:AF334206.1,INSD:BT026517.1"
                     /inference="Similar to RNA sequence,
                     EST:INSD:DR750187.1,INSD:DR350305.1,INSD:DR225222.1,
                     INSD:ES148762.1,INSD:ES150720.1,INSD:DR225221.1,
                     INSD:ES001012.1,INSD:DR384053.1,INSD:ES005968.1,
                     INSD:ES088346.1,INSD:EL982026.1,INSD:AV785099.1,
                     INSD:ES143521.1,INSD:BP650811.1,INSD:DN604474.1,
                     INSD:DR350304.1,INSD:DR260503.1,INSD:EL987210.1,
                     INSD:EL997657.1,INSD:ES163518.1,INSD:ES101562.1,
                     INSD:DR260508.1,INSD:ES152551.1,INSD:BP644492.1,
                     INSD:AV820566.1,INSD:AV823993.1,INSD:ES092060.1,
                     INSD:DR260509.1"
                     /note="Basic-leucine zipper (bZIP) transcription factor
                     family protein ABA INSENSITIVE 5 (ABI5); CONTAINS InterPro
                     DOMAIN/s: Basic-leucine zipper (bZIP) transcription factor
                     (InterPro:IPR004827), bZIP transcription factor, bZIP-1
                     (InterPro:IPR011616); BEST Arabidopsis thaliana protein
                     match is: abscisic acid responsive element-binding factor
                     1 (TAIR:AT1G49720.1); Has 3780 Blast hits to 3404 proteins
                     in 335 species: Archae - 4; Bacteria - 363; Metazoa - 820;
                     Fungi - 233; Plants - 1594; Viruses - 12; Other Eukaryotes
                     - 754 (source: NCBI BLink)."
                     /db_xref="GeneID:818199"
                     /db_xref="TAIR:AT2G36270"
```

Mascot: http://www.matrixscience.com/

Supplementary Figure 5. Analysis of *in vitro* ABI5 *S*-nitrosylation.

Protein sequence using protein view-MASCOT search, including observed mass, expected mass, calculated mass, Mascot score, expectation score and rank, and peptide sequence. Nominal mass (Mr).

a

Mascot Search Results

Peptide View

MS/MS Fragmentation of QGSLTLPAPLCR
Found in gi|18404091 in NCBInr, protein abscisic acid-insensitive 5 [Arabidopsis thaliana]

Match to Query 320: 1682.965448 from(842.490000,2+) intensity(304169.0000) index(316)
Title: Cmpd 317, +MSn(842.5), 49.5 min
Data file MCR3_sin reducir_30%_RC6_01_5646.mgf

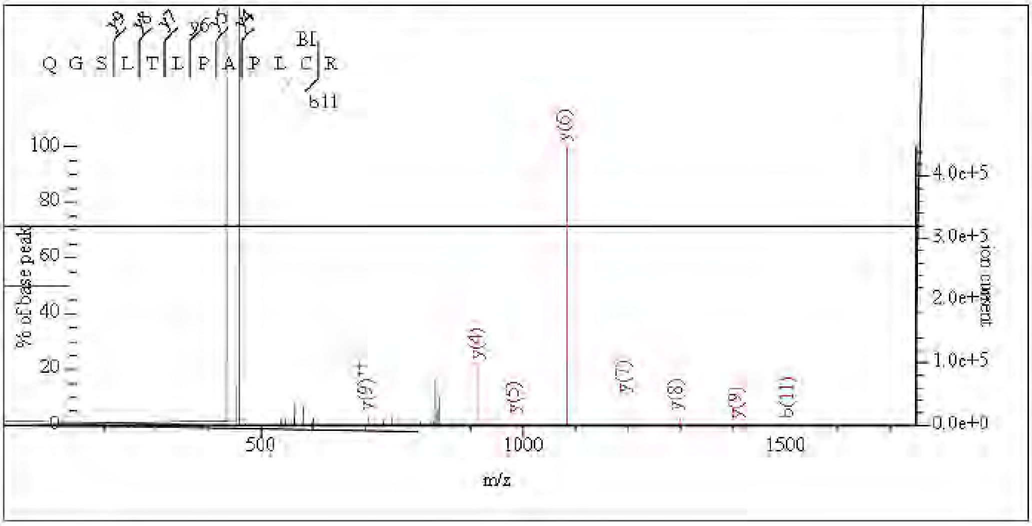

- Help
- Export as SVG

Pan leftZoom inZoom outUndo pan or zoomZoom reset 148.93 to 1750 Zoom to rangePan right

Label all possible matches ○ Label matches used for scoring ⊙

Monoisotopic mass of neutral peptide Mr(calc): 1682.8670
Variable modifications:
C11 : Biotin-HPDP (C)
Ions Score: 48 **Expect:** 0.13
Matches : 9/114 fragment ions using 13 most intense peaks (help)

#	b	b^{++}	b^{*}	b^{*++}	b^{0}	b^{0++}	Seq.	y	y^{++}	y^{*}	y^{*++}	y^{0}	y^{0++}	#
1	129.0659	65.0366	112.0393	56.5233			Q							12
2	186.0873	93.5473	169.0608	85.0340			G	1555.8157	778.4115	1538.7892	769.8982	1537.8052	769.4062	11
3	273.1193	137.0633	256.0928	128.5500	255.1088	128.0580	S	1498.7943	749.9008	1481.7677	741.3875	1480.7837	740.8955	10
4	386.2034	193.6053	369.1769	185.0921	368.1928	184.6001	L	***1411.7622***	706.3848	1394.7357	697.8715	1393.7517	697.3795	9
5	487.2511	244.1292	470.2245	235.6159	469.2405	235.1239	T	***1298.6782***	649.8427	1281.6516	641.3294	1280.6676	640.8374	8
6	600.3352	300.6712	583.3086	292.1579	582.3246	291.6659	L	***1197.6305***	599.3189	1180.6039	590.8056			7
7	697.3879	349.1976	680.3614	340.6843	679.3774	340.1923	P	***1084.5464***	542.7769	1067.5199	534.2636			6
8	768.4250	384.7162	751.3985	376.2029	750.4145	375.7109	A	***987.4937***	494.2505	970.4671	485.7372			5
9	865.4778	433.2425	848.4512	424.7293	847.4672	424.2373	P	***916.4565***	458.7319	899.4300	450.2186			4
10	978.5619	489.7846	961.5353	481.2713	960.5513	480.7793	L	819.4038	410.2055	802.3772	401.6923			3
11	1509.7626	755.3850	1492.7361	746.8717	1491.7521	746.3797	C	***706.3197***	353.6635	689.2932	345.1502			2
12							R	175.1190	88.0631	158.0924	79.5498			1

Error Distribution Error Distribution (ppm)

NCBI **BLAST** search of QGSLTLPAPLCR
(Parameters: blastp, nr protein database, expect=20000, no filter, PAM30)
Other BLAST web gateways

b

All matches to this query

Score	Mr(calc)	Delta	Sequence
47.9	1682.8670	0.0984	QGSLTLPAPLCR
24.0	1682.8951	0.0703	SSAIQLPAGKSPVGDTR
21.9	1682.8450	0.1204	CYKIGSSTFPLSGAPR
21.6	1682.8886	0.0769	LSCSLSDPSPPLRRR
19.0	1682.7990	0.1665	GTRHMNALPCR
15.4	1682.8965	0.0690	QHGEIRFIGRTDVR
15.3	1682.8515	0.1139	NGLTTLFKEDPSAYK
14.2	1683.0155	-0.0501	RVGIVGLGSTGSLIAKR
14.1	1682.9328	0.0326	HRSHPITSAPLRPSK
13.9	1682.8587	0.1067	EGETTIGEVAPATPRR

Supplementary Figure 6. Analysis of *in vitro* ABI5 *S*-nitrosylation.

a, Peptide view-MASCOT search showing the MS/MS spectrum.

b, Peptide view-MASCOT search showing all matched ions. Nominal mass (Mr).

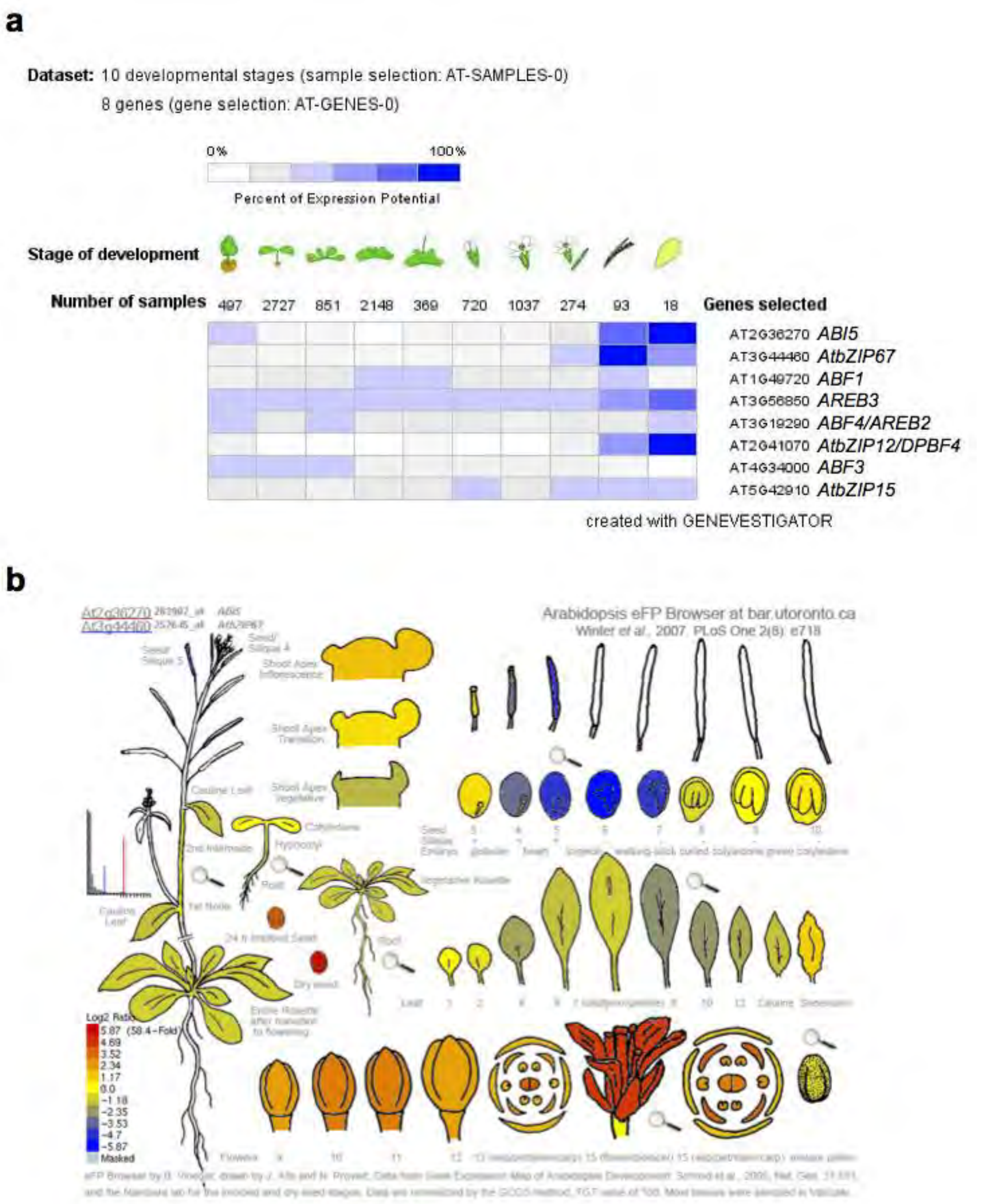

Supplementary Figure 7. Expression pattern of *ABI5* and *ABI5-like* genes.

a, Gene expression profiles of *ABI5* and *ABI5-like* genes relative to the developmental map using the Meta-Analyzer platform from Genevestigator software (http://www.genevestigator.ethz.ch) reveals that expression of *ABI5* (At2g36270) was present in seeds[3].

b, Comparison of the transcription levels of *ABI5* (At2g36270) vs. *AtbZIP67* (At3g44460) in different plant tissues, based on data obtained using the eFP browser (http://bar.utoronto.ca).

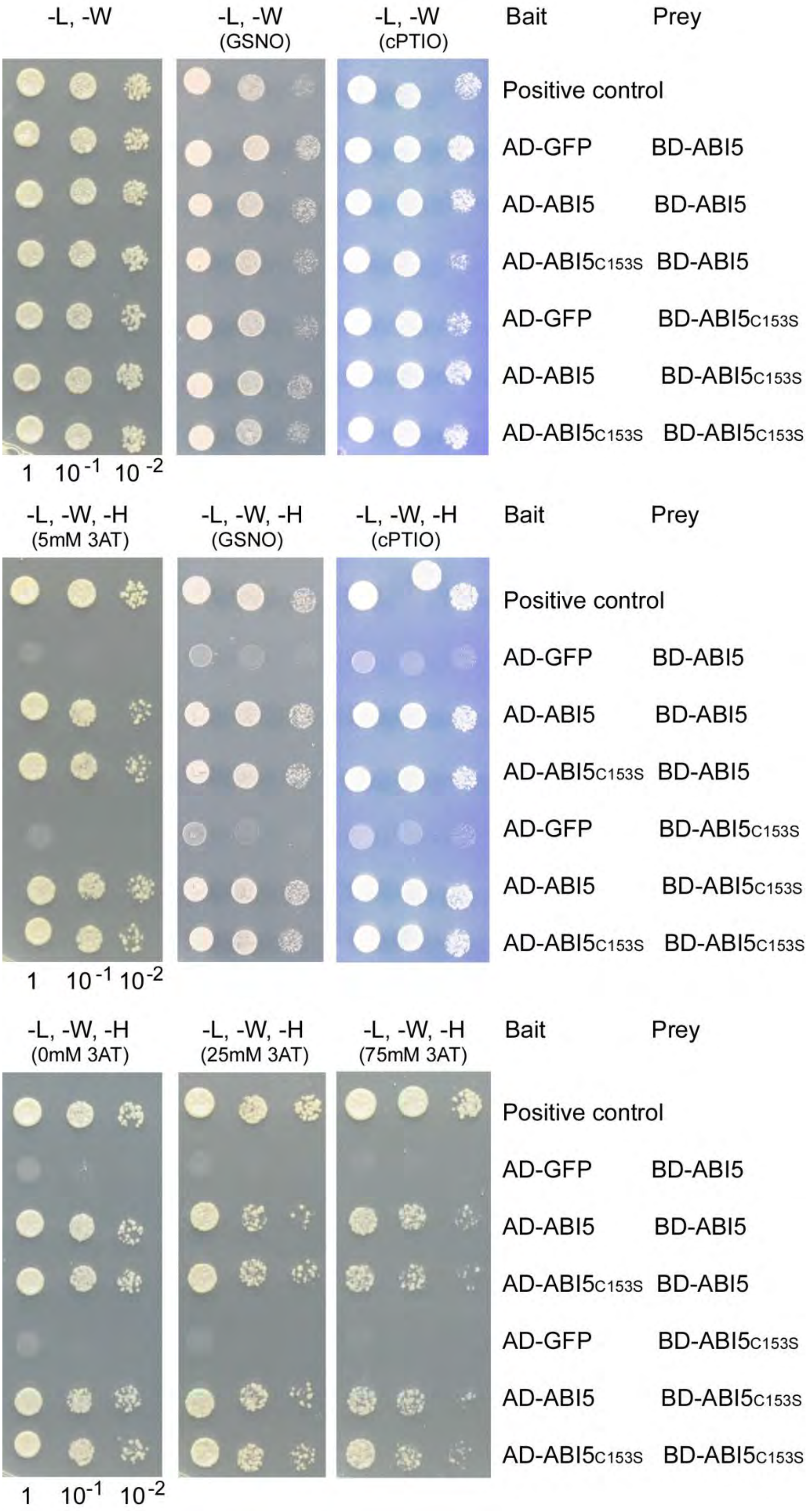
-L, -W
-L, -W (GSNO)
-L, -W (cPTIO)
Bait
Prey
Positive control
AD-GFP BD-ABI5
AD-ABI5 BD-ABI5
AD-ABI5C153S BD-ABI5
AD-GFP BD-ABI5C153S
AD-ABI5 BD-ABI5C153S
AD-ABI5C153S BD-ABI5C153S
1 10-1 10-2
-L, -W, -H (5mM 3AT)
-L, -W, -H (GSNO)
-L, -W, -H (cPTIO)
Bait
Prey
Positive control
AD-GFP BD-ABI5
AD-ABI5 BD-ABI5
AD-ABI5C153S BD-ABI5
AD-GFP BD-ABI5C153S
AD-ABI5 BD-ABI5C153S
AD-ABI5C153S BD-ABI5C153S
1 10-1 10-2
-L, -W, -H (0mM 3AT)
-L, -W, -H (25mM 3AT)
-L, -W, -H (75mM 3AT)
Bait
Prey
Positive control
AD-GFP BD-ABI5
AD-ABI5 BD-ABI5
AD-ABI5C153S BD-ABI5
AD-GFP BD-ABI5C153S
AD-ABI5 BD-ABI5C153S
AD-ABI5C153S BD-ABI5C153S
1 10-1 10-2

Supplementary Figure 8. ABI5 Cys153 mutation does not abolish protein homodimerization. Yeast two-hybrid assay for ABI5 protein homodimerization. Two-hybrid interaction test of ABI5 (wild-type) and ABI5C153S (mutated version). Upper, control plates. Middle and right, test plates (-His) and (-His, +3-Amino-Triazole), respectively. For the constructs, the ABI5 and ABI5C153S were fused to the Gal4 DNA-binding domain (BD) and Gal4 activation domain (AD) as indicated.

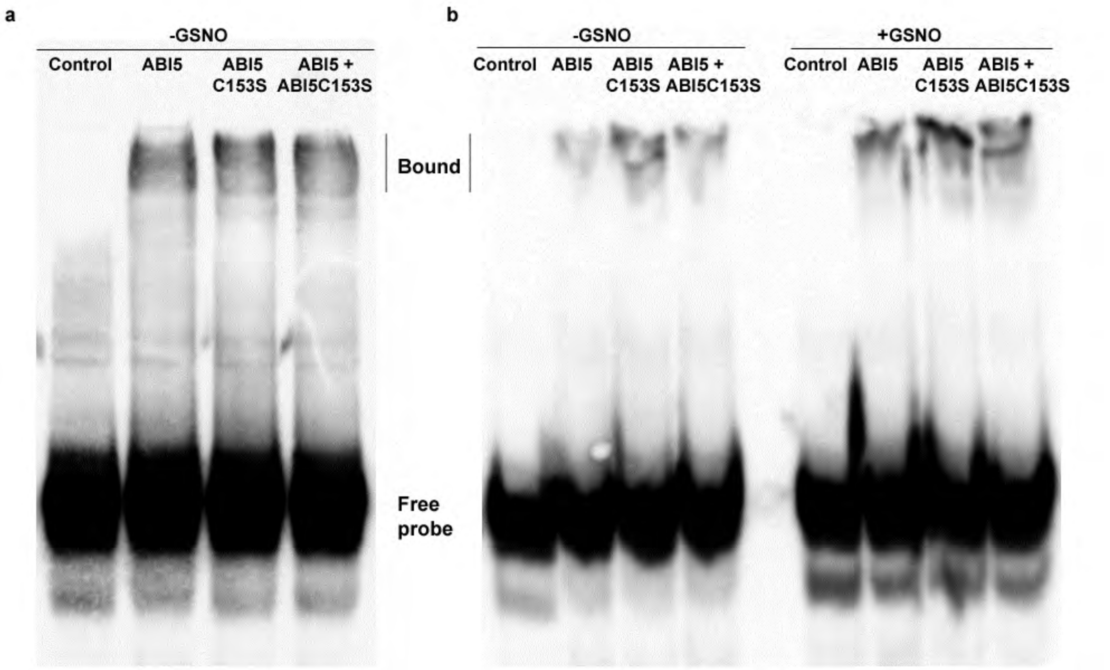

Supplementary Figure 9. ABI5 Cys153 mutation and *S*-nitrosylation do not abolish DNA binding.

a, Electrophoretic mobility shift assay (EMSA) of ABI5 (wild-type) and ABI5C153S (mutated version) binding to DNA fragments containing the ABRE motif under reducing conditions.

b, EMSA of ABI5 (wild-type) and ABI5C153S (mutated version) binding to DNA fragments containing the ABRE motif under non-reducing conditions and in the absence and presence of GSNO (1 mM).

See Methods for a description of the probe. (Control) ABRE-box incubated without recombinant protein.

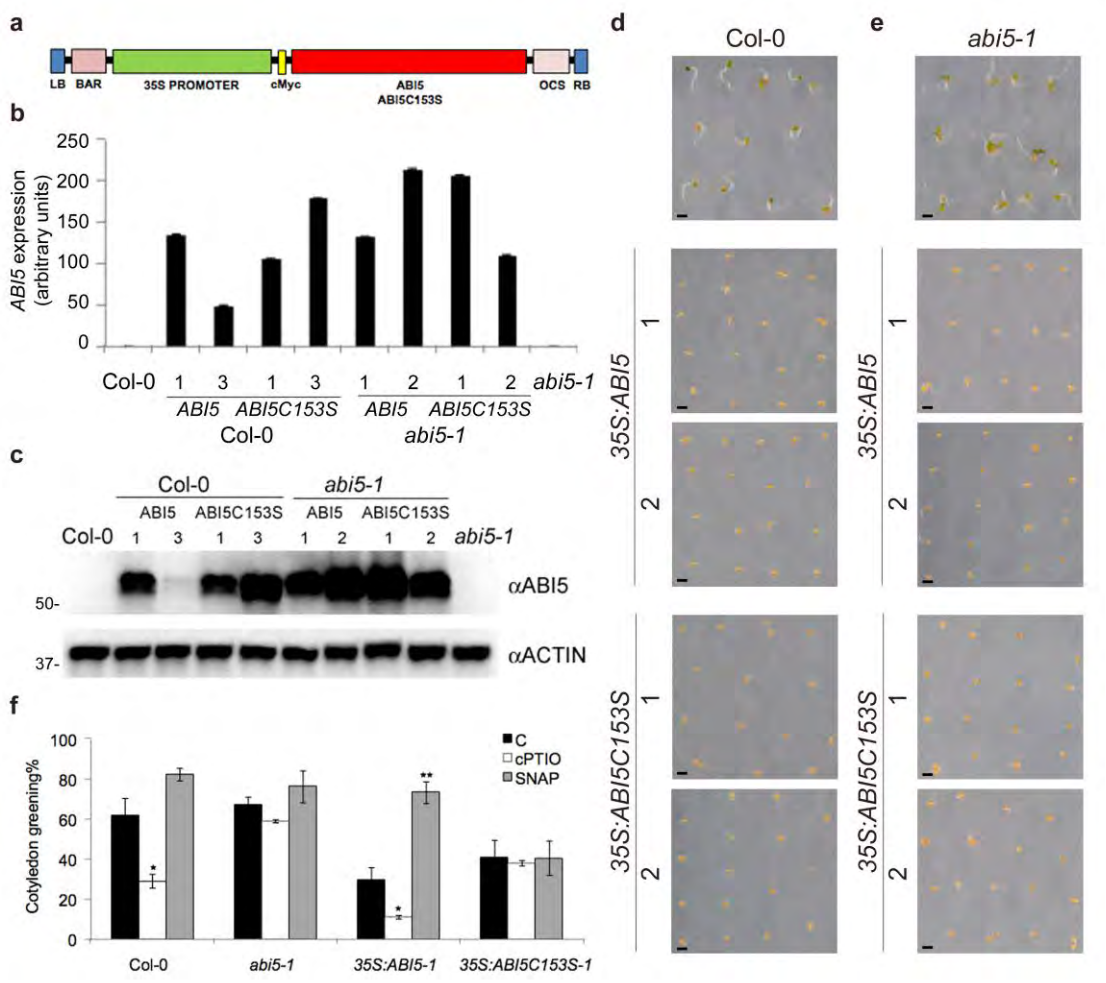

Supplementary Figure 10. Generation and molecular analysis of *35S:ABI5* and *35S:ABI5C153S* transgenic lines.

a, Construct used to generate the transgenic lines.

b, Q RT-PCR analysis of *ABI5* expression levels in Col-0, the *abi5-1* background and *35S:ABI5* and *35S:ABI5C153S* transgenic lines.

c, ABI5 levels in *35S:ABI5* and *35S:ABI5C153S* transgenic plants. Immunoblot analysis of *in vivo* ABI5 protein levels in *35S:ABI5* and *35S:ABI5*C153 seedlings. Actin protein levels were also determined as a loading control.

d, Seed-specific phenotypes of *35S:ABI5* and *35S:ABI5C153S* lines. ABA-hypersensitive inhibition of germination and post-germinative growth in two *35S:ABI5* and *35S:ABI5C153S* lines as compared to wild-type plants. Photographs show wild-type (Col-0) and two *35S:ABI5* and *35S:ABI5C153S* independent lines (1, 2) in medium supplemented with 0.2 µM ABA 4 days after sowing. Scale bars 1 mm.

e, Restoration of *abi5-1* mutation by both *35S:ABI5 and 35S:ABI5C153S* constructs. ABA-hypersensitive inhibition of germination and seedling establishment in *35S:ABI5* and *35S:ABI5C153S* lines compared to wild-type and *abi5-1* mutant plants.

Photographs show wild-type *abi5-1* and two *35S:ABI5* and *35S:ABI5C153S* independent lines (1, 2) in medium supplemented with 0.2 µM ABA 4 days after sowing. Scale bars 1 mm.

f, NO-insensitivity during seedling establishment in *35S:ABI5C153S* lines as compared to *35S:ABI5* plants. NO donor and scavenger effect in seedling growth of wild-type (Col-0), *abi5-1*, *35S:ABI5* and *35S:ABI5C153S* lines grown for 3 days on MS agar plates untreated (Control), or supplemented with 300 µM of the NO-donor SNAP and 100 µM of the NO-scavenger cPTIO. Values represent the mean ±SE (n=3). Asterisks indicate significant differences compared with Control (t-test, *P<0.05, **P<0.01).

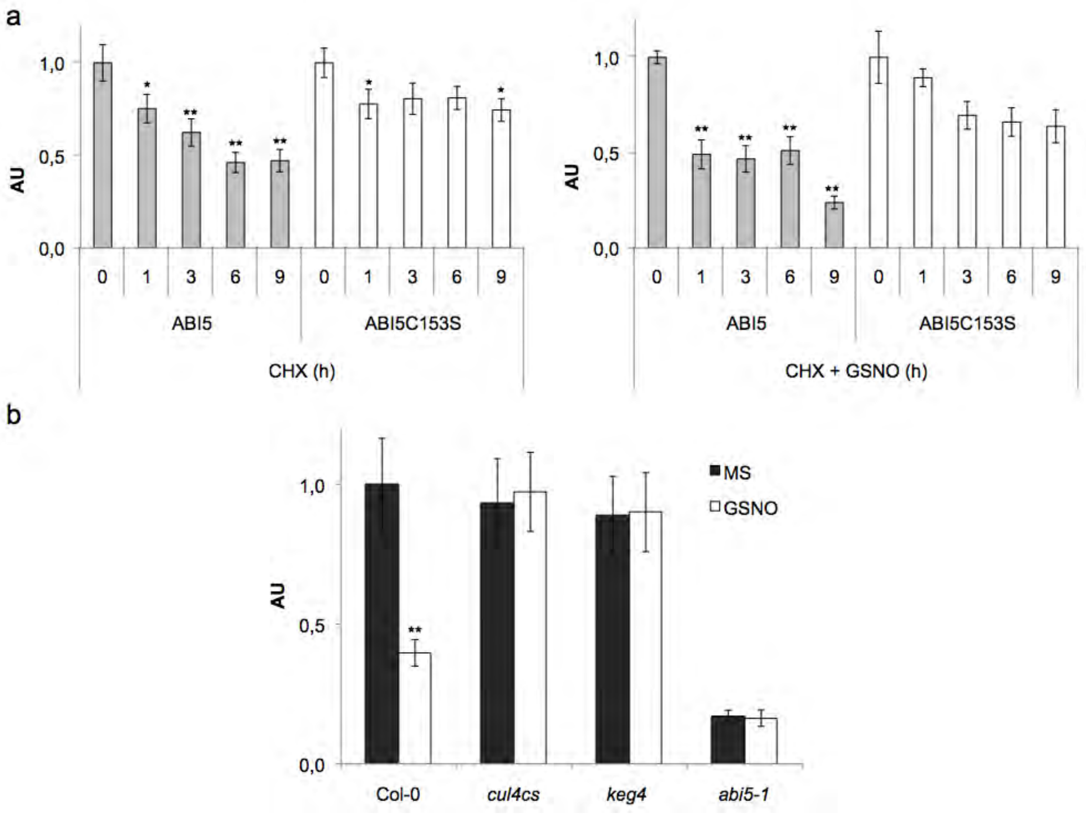

Supplementary Figure 11. Quantitative data of NO-promoted ABI5 degradation.

a, Quantitative data of immunoblot analysis of ABI5 (wild-type) and ABI5C153S (mutated version) degradation by application of GSNO (500 µM) in the presence of cycloheximide (CHX, 1 mM). Values represent the mean ±SE (*n*=3) and normalized against actin, as shown in Fig. 4a. Asterisks indicate significant differences compared with 0 hours (*t*-test, **P*<0.05, ***P*<0.01).

b, Quantitative data of immunoblot analysis of CUL4 and KEG involvement in NO-promoted ABI5 degradation. Values represent the mean ±SE (*n*=3) and normalized against actin, as shown in Fig. 4b. Asterisks indicate significant differences compared with 0 hours (*t*-test, **P*<0.05, ***P*<0.01).

AU, arbitrary units.

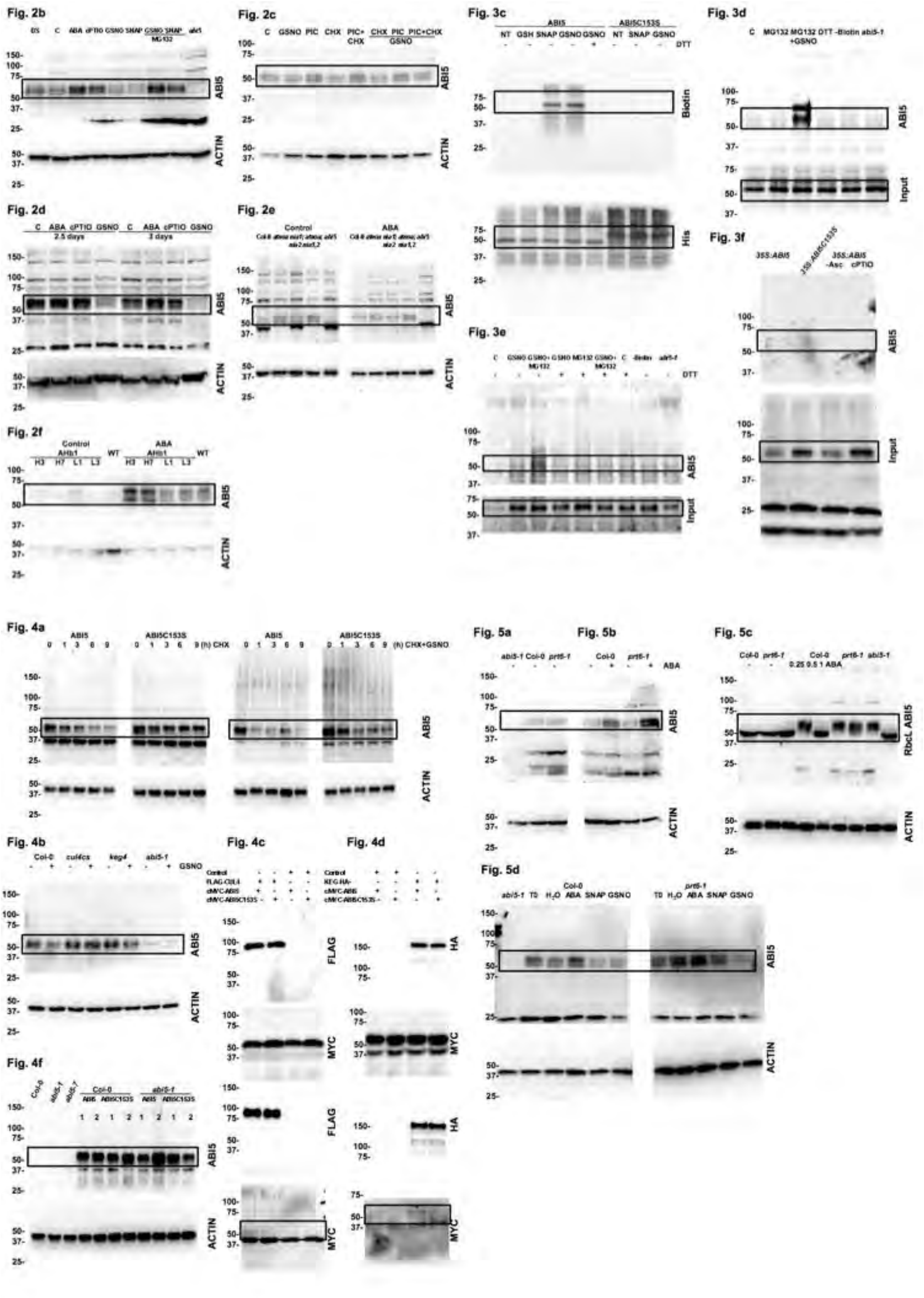

Supplementary Figure 12. Uncropped scans of the most important immunoblot results. Black boxes highlight lanes used in figures.

Supplementary References

1. Xue, Y., Liu, Z., Gao, X., Jin, C., Wen, L., Yao, X. & Ren, J. GPS-SNO: Computational prediction of protein *S*-nitrosylation sites with a modified GPS algorithm. *Plos One* **5**, e11290 (2010).
2. Lee, T.Y., Chen, Y.J., Lu, C.T., Ching, W.C., Teng, Y.C., Huang, H.D. & Chen, Y.J. dbSNO: a database of cysteine *S*-nitrosylation. *Bioinformatics* **28**, 2293-2295 (2012).
3. Zimmermann, P., Hirsch-Hoffmann, M., Henning, L. & Gruissem, W. GENEVESTIGATOR. Arabidopsis microarray database and analysis toolbox. *Plant Physiol.* **136**, 2621-2632 (2004).
